# Design, Synthesis, and Antiproliferative Activity of Novel Neocryptolepine–Rhodanine Hybrids

**DOI:** 10.3390/molecules27217599

**Published:** 2022-11-05

**Authors:** Mohamed El-Bahnsawye, Mona K. Abo Hussein, Elshaymaa I. Elmongy, Hanem Mohamed Awad, Aliaa Abd El-Kader Tolan, Yasmine Shafik Moemen, Ahmed El-Shaarawy, Ibrahim El-Tantawy El-Sayed

**Affiliations:** 1 Chemistry Department, Faculty of Science, Menoufia University, Shebin El-Kom 32511, Egypt; 2 Clinical Microbiology and Immunology Department, National Liver Institute, Menoufia University, Shebin El-Kom 32511, Egypt; 3 Department of Pharmaceutical Sciences, College of Pharmacy, Princess Nourah Bint Abdulrahman University, P.O. Box 84428, Riyadh 11671, Saudi Arabia; 4 Tanning Materials and Leather Technology Department, National Research Centre, Dokki, Giza 12622, Egypt; 5 Clinical Pathology Department, National Liver Institute, Menoufia University, Shebin El-Kom 32511, Egypt

**Keywords:** neocryptolepine, alkaloids, rhodanine, hybrids, docking, pharmacokinetics

## Abstract

A series of novel neocryptolepine–rhodanine hybrids (**9a,b**, **11a**–**d**, **14**, and **16a,b**) have been synthesized by combining neocryptolepine core **5** modified at the C-11 position with rhodanine condensed with the appropriate aryl/hetero aryl aldehydes. Based on these findings, the structures of the hybrids were confirmed by spectral analyses. By employing the MTT assay, all hybrids were tested for their in vitro antiproliferative activity against two cancer cell lines, including MDA-MB-231 (human breast) and HepG-2 (hepatocellular carcinoma). Interestingly, the IC_50_ values of all hybrids except **9b** and **11c** showed activity comparable to the standard anticancer drug, 5-fluorouracil, against HepG-2 cancer cells. Furthermore, the cytotoxicity of all the synthesized hybrids was investigated on a normal skin human cell line (BJ-1), and the results showed that these compounds had no significant cytotoxicity toward these healthy cells at the highest concentration used in this study. This study also indicated that the active hybrids exert their cytotoxic activity via the induction of apoptosis. A molecular docking study was used to shed light on the molecular mechanism of their anticancer activity. The docking results revealed that the hybrids exert their mode of action through DNA intercalation. Furthermore, in silico assessment for pharmacokinetic properties was performed on the most potent compounds, which revealed candidates with good bioavailability, high tolerability with cell membranes, and positive drug-likeness values.

## 1. Introduction

Cancer is one of the primary causes of death worldwide and the pursuit of novel, clinically useful anticancer agents is, therefore, one of the top priorities for medicinal chemists. The advancement of selective medications that target malignant tumor cells without damaging normal cells while minimizing the danger of adverse effects is a critical goal of cancer chemotherapy [1]. This has spurred extensive research to identify novel chemo-preventative drugs that are very effective and have fewer adverse effects.

Natural products are always the major source of molecular targets, mostly because of their wide structural diversity; they are a reliable source for the production of pharmaceutically relevant lead molecules [2]. The capability of natural chemicals to become lead compounds has been shown by the existence of Taxol, an anticancer medication that has been used to treat a variety of malignancies [3]. *CryptolePis sanguinolenta* [4] is a promising natural plant whose roots are utilized in traditional medicine in Central and West African countries [5] to treat various ailments including malaria, diabetes, and inflammatory disorders [6,7].

**Figure 1:**
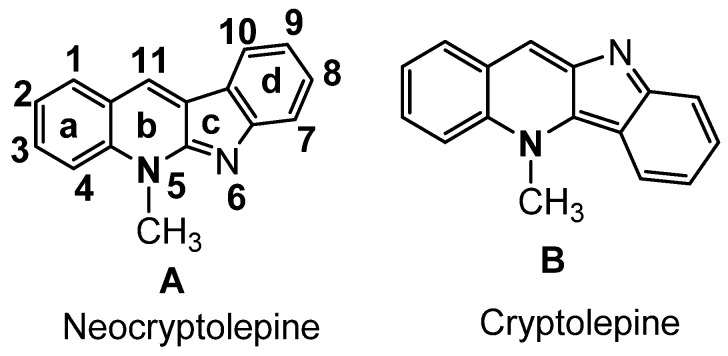
Indoloquinoline from *CryptolePis Sanguinolenta*.

**Figure 2:**
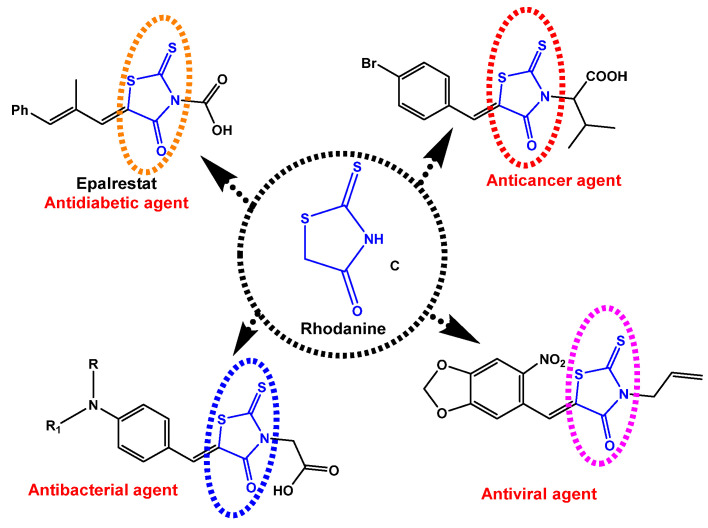
Some biological applications of rhodanine structures C.

The two common extracted active constituents of the natural plant are neocryptolepine A and its regio-isomer, cryptolePine B indoloquinoline alkaloids (Figure 1). neocryptolepine A was shown to be substantially less cytotoxic than cryptolePine, enabling the development of novel lead compounds from its derivatives [8]. The alkaloid neocryptolepine A “5-methyl-indolo [2,3-b] quinoline” had shown antibacterial [9,10,11], antimalarial [12,13], antiproliferative [14,15], antischistosomicidal, and antiplasmodial activity [16,17,18,19,20]. Additional experimental results have indicated that the antiproliferative activity of A was due to the intercalation between the DNA base pairs, followed by enzymatic inhibition of topoisomerase I and II, thereby inhibiting DNA synthesis, replication, and transcription in host cancer cells [8]. A previous structure–activity relationships (SARs) study revealed that introducing an amino side chain to the neocryptolepine core enhanced the antiproliferative activity. The rationale behind introducing the amino side chains into the core of neocryptolepine was justified due to their indispensable role in antiproliferative activity. On the other hand, rhodanine, 2-thioxo-4-thiazolidone C, and its analogs (Figure 2) have a wide range of biological activities such as antidiabetic [21], antiviral [22,23], anti-inflammatory [24], antimicrobial [25], antimalarial [26], antifungal [27,28], anti-HIV [29], and anticancer agents [30,31,32,33,34,35,36,37].

The present study aims at construction of urgently required highly efficient new molecular entity as bio-active leads using rational design strategy. These leads seem highly needed due to the limited application and side effects of the already used anticancer drugs. They may help to resolve current issues as side effects in addition to cell resistance to tumor as well as low efficacy. These wide medical diversity of neocryptolepine and rhodanine as well as their corresponding analogues prompted us to explore conjugated lead combing both pharmacophoric moieties in one hybrid molecular target in search for synergistic effect on biological activity. The aforementioned finding encouraged us to further modification to the side chains at the C-11 position by conjugation with a variety of pharmacologically active moieties such as rhodanine. It is clear that a vibrant drug discovery Pipeline is needed in the anticancer field so as to gurantee the availability of new compounds that meet the desired target product profiles with the potential to feed the preclinical Pipeline. One of the most challenging aspects is the early innovation stage of discovering new lead series of molecules. Herein, novel neocryptolepine-rhodanine hybrids will be synthesised and evaluated for their antiproliferative activity against liver and breast cancer cell lines.

## 2. Results

### 2.1. Chemistry

#### 2.1.1. Synthesis of 11-Chloroneocryptolepine **5**

**Scheme 1:**
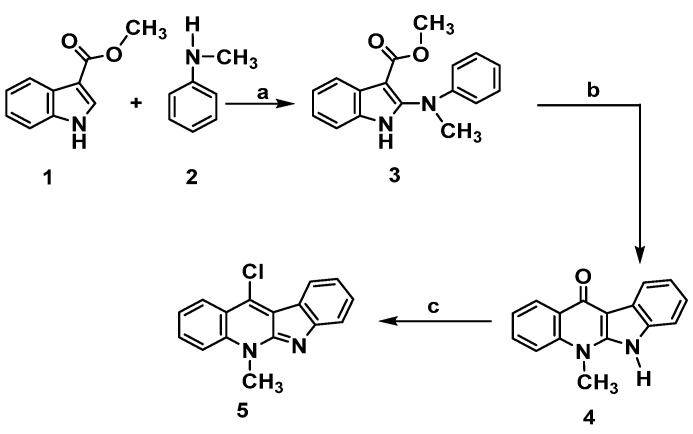
Synthesis of neocryptolepine **5**. Reagents and conditions: (a) *N*-chlorosuccinimide, 1,4-dimethylPiperazine, CH_2_Cl_2_, 0 °C, 2 h. b. Trichloroacetic acid, room temperature, 2 h. (b) Diphenyl ether, reflux, 3 h. (c) POCl_3_, toluene, reflux, 12 h.

The preparation of the key intermediate 11-chloroneocryptolepine **5** required for the diversification of the parent natural compound has been executed as described in Scheme 1 [15,16,19,22]. This approach leads to synthesize new analogues by incorporating different substitutions at structure A- ring b. The methodology used 1H-methyl indole-3-carboxylate **1** and N-methylaniline **2** as a lead to start synthesis of the targeted compound **5.** Synthesis of the intermediate, phenyl amino indolo carboxylate derivative **3**, was obtained by chlorination using trichloro acetate salt of N-methylaniline **2** just upon the reaction with N-chlorosuccinimide (NCS) in the presence of 1,4-dimethylPiperazine. Cyclization of the result product was performed in boiling diphenyl ether to give **4**. Dehydroxy-chlorination was done with POCl_3_ to produce **5**, as shown in Scheme 1. The choice of using N-methylamine instead of aniline was based on our previous work (**16a,b**) that revealed issues upon using free aniline as low yield, solubility as well as long reaction time associated with the methylation step. Employing N- methyl anilines greatly facilitate the reaction by reducing the reaction time and improving the result yield. Moreover, the target compound 5 was prepared in good yield with less steps. Scheme 1.

**Scheme 2:**
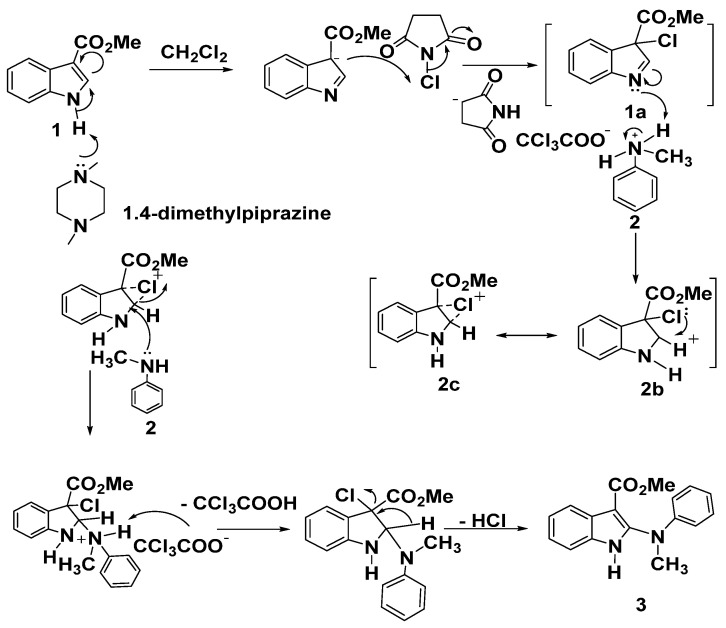
A plausible mechanism for the formation of product **3**.

Mechanism for synthesis of compound **3** was illustrated in Scheme 2. Conversion of **1** into the intermediate 3-chloro-indolenine **1a** using N-chlorosuccinimide (NCS) in the presence of a base was the generally accepted, then cyclic carbenium ion **2b** was obtained upon protonation of **1a**, possibly stabilized by the cyclic chloronium ion intermediate **2c**, finally, nucleophilic attack of aniline nitrogen to afford **3** as dePicted below in Scheme 2.

**Scheme 3:**
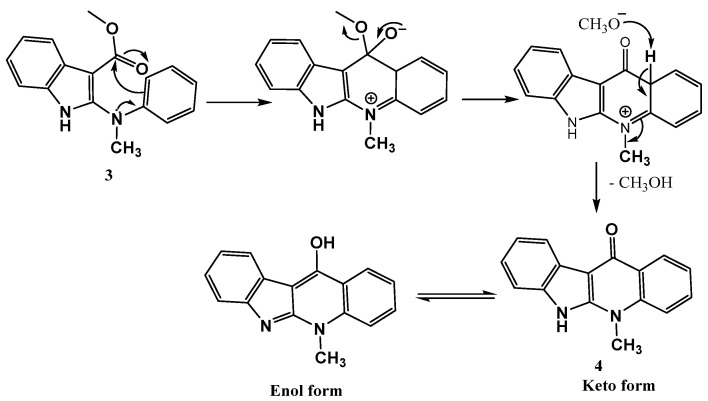
Formation and mechanism of cyclic ketone **4**.

Furthermore, a mechanism was proposed for preparing the structure **4** included the intramolecular nucleophilic substitution at the ester carbonyl carbon on the indole core via the electrophilic acylation of the nucleophilic benzene ring followed by the elimination of methanol to afford the indoloquinolone **4**, as dePicted in the following Scheme 3.

**Scheme 4:**
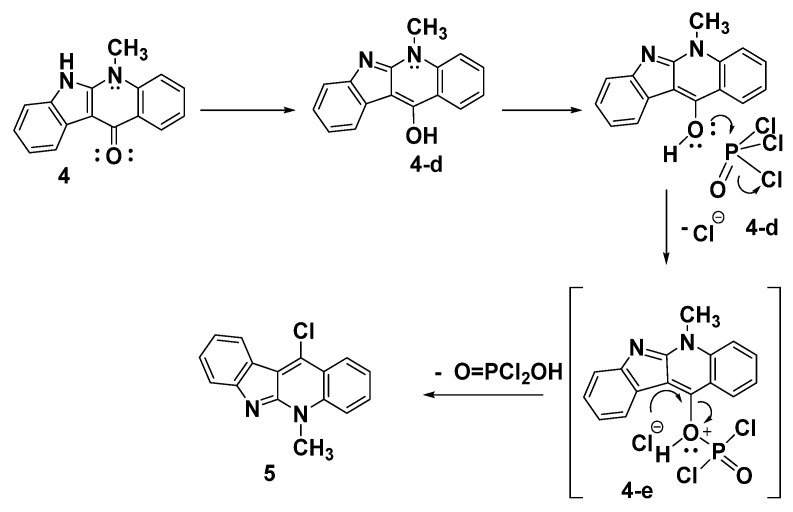
A possible method for the manufacture of compound **5**.

A proposed mechanism for synthesis of the target product **5** upon reacting the indoloquinoline derivative **4** with POCl_3_ is illustrated in Scheme 4. The reaction involves the tautomerization of indoloquinoline **4** to form the enol form **4d.** The nucleophilic attack of the hydroxy group of **4d** on the electrophilic phosphorus of POCl_3_, followed by the elimination of the chloride ion, led to the formation of the intermediate **4e**. The dehydroxy-chlorination of **4e** gives the corresponding 11-chloroneocryptolepine **5**, as shown in Scheme 4.

#### 2.1.2. Synthesis of 11-Aminoalkyleneamino Neocryptolepines **7a,b**

**Scheme 5:**
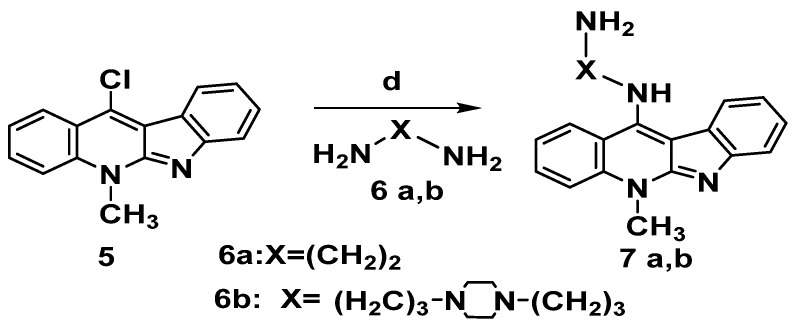
Synthesis of 11-aminoalkylene aminoneocryptolepine **7a**,**b**. Reagents and conditions: (d) Excess alkylene bisamine **6a**,**b**, neat, reflux 5–10 min.

Incorporation of amino-alkylene amine side chain is expected to improve DNA intercalation binding capacity via hydrogen bonding motifs. Hence, to boost the electrostatic interactions with the phosphate group in the DNA backbone structure, we made integeration between various chains and the ionizable amine, in each case, the compounds were designed to include structural properties that could boost DNA binding affinity, selectivity to cancer cells and cell viability as well. Choosing the indoloquinoline scaffold was based on its well-documented DNA binding capabilities and intercalation-mediated DNA binding interactions [8]. As a result, the critical intermediate 11-chloroneocryptolepines **5** was employed to diversify the neocryptolepine core at the C-11 position. Thus, the reaction of **5** with a large excess of alkylene bis amine **6a**,**6b** (without solvent) and heating for 5 to 10 min produced the corresponding 11-aminoalkylene amino-neocryptolepine analogs **7a**,**b** smoothly and in very good yields, as illustrated in Scheme 5. It is also worth noting that during the course of the reaction, no dimeric product was observed, though a dimeric product is anticipated. To avoid this problem, the reaction condition was optimized by using a large excess of bis-amines, conducting the reaction without solvent (a neat reaction). In addition, under these optimal conditions, the amination step was completed in a very short time (minutes) and no dimeric product was found as monitored by TLC.

**Scheme 6:**
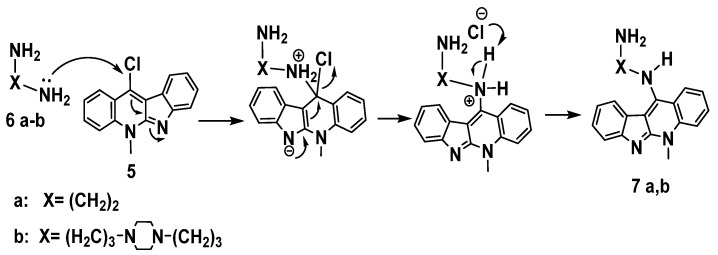
Proposed mechanism of aromatic nucleophilic substitution for the synthesis of **7a**,**b**.

Nucleophilic aromatic substitution (SNAr) reaction mechanism for synthesis of derivatives 7a-b in which, substitution of the chlorine atom by the amino group was done at the unsaturated sp^2^ C-11 position, Scheme 6.This reaction proceeds through formation of a resonance-stabilized anion with a new C– N bond upon addition of the amino group (: Nu–) followed by elimination of HCl to yield **7a-b** as in the mechanism below, Scheme 6.

#### 2.1.3. Synthesis of Neocryptolepine–Rhodanine Hybrids

**Scheme 7:**
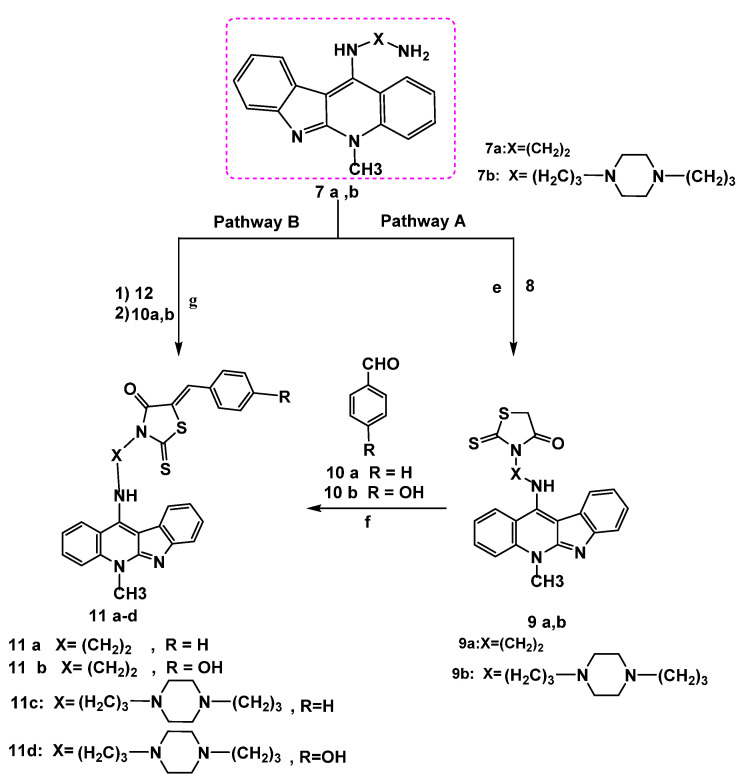
Reagents and conditions: Pathway A: (e) ethyl bromoacetate **8**, CS_2_, acetonitrile r.t, 12–24 h. (f) NaOAc, glacial acetic acid, stirring, reflux 6–8 h. Pathway B: (g) ethyl chloroacetate **12** (1 mmol), r.t 30 min–1 h, CS_2_ (2 mmol) in (2 mL) DMF, KOH (3 mmol) and **10 a**,**b** (1 mmol), r.t overnight.

**Scheme 8:**
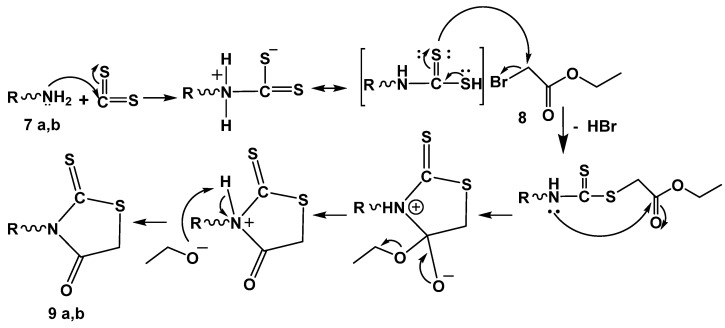
Mechanism of rhodanine ring formation **9a**,**b**. R = alkylene amino neocryptolepine.

To improve the anticancer activity of the neocryptolepine core structure, the synthesis of novel neocryptolepine conjugated with rhodanine moiety was elaborated. To that end, a three-component reaction of amines **7a**,**b**, carbon disulfide, and ethylbromo acetate **8** in acetonitrile at room temperature provided the cyclized intermediates **9a**,**b** via an addition–elimination reaction at room temperature, as shown in Scheme 7 and Scheme 8.

**Scheme 9:**
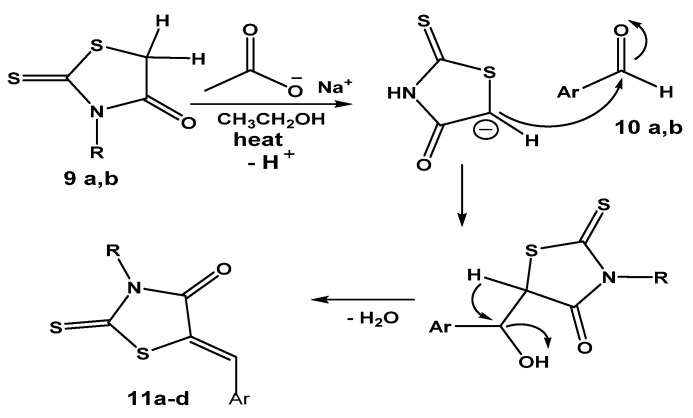
Plausible mechsm of hybrids **11a**–**d** formation via Knoevenagel condensation reaction with aldehydes **10a**,**b**. where R = alkylene amino neocryptolepine, Ar = Ph or 4-hydroxyphenyl.

Furthermore, the carbon–carbon forming step of the Knoevenagel reaction was achieved [33] as a result of a nucleophilic addition between aldehydes **10a**,**b** and an active methylene group of a rhodanine ring in **9a**,**b** in the presence of sodium acetate in glacial acetic acid as a base. The deprotonation of the C-H bond by base was followed by the nucleophilic addition of the resulting anionic carbon of the rhodanine ring after deprotonation to the electrophilic carbon of the carbonyl group in the aldehyde **10a**,**b**. Further spontaneous dehydration with the formation of the exocyclic double bond afforded the expected hybrids **11a**–**d** in good yields, as shown in Scheme 9.

#### 2.1.4. Synthesis of 11-Aminoneocryptolepine–Rhodanine Hybrids **16a**,**b**

**Scheme 10:**
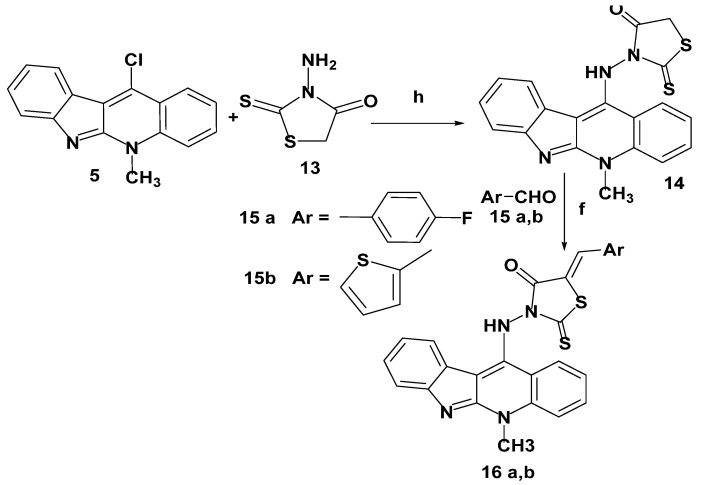
Synthesis of neocryptolepine–rhodanine hybrids **16a**,**b**. Reagents and conditions: (h) DMF, Et_3_N, reflux 1–4 h.(f) Sodium acetate, glacial acetic acid, stirring with reflux, 12–24 h.

A mixture of 11-chloroneocryptolepine **5** and 3-aminorhodanine **13,** in presence of dimethyl formamide (DMF) and triethylamine as a base, was refluxed to afford a new hybrid **14** via the nucleophilic aromatic substitution (S_NAr_) reaction mechanism. Further assembling structures bearing neocryptolepine-5-arylidine rhodanine hybrids **16a**,**b** were obtained via the Knoevenagel condensation reaction with appropriate heterocyclic aldehydes **15a**,**b** in the presence of sodium acetate and glacial acetic acid, as given in Scheme 10. It is worthwhile to note that the new arylidine–rhodanine hybrids **16a**,**b** were tyPically obtained as a single geometric isomer, as elucidated by ^1^H-NMR spectral analysis, and the geometry around the exocyclic double bond of the rhodanine ring was assigned as the *Z* isomer and consistent with closely similar reported data [36]. The chemical shift of the olefinic proton of the (*Z*)–isomer ranged from 7.39 to 7.94 ppm, whereas the (*E*)–isomer showed a chemical shift of 6.78–7.01 ppm. As a result of our obtained ^1^H-NMR results, the synthesized hybrids **16a**,**b** proved to be (*Z*)-isomers.

All the newly synthesized compounds were characterized by IR, ^1^HNMR, ^13^C NMR, and mass spectroscopy. This showed results in good agreement with the proposed chemical structures, and the known compounds showed data consistent with the literature data. For the new hybrids, FTIR spectroscopy was used for characterizing the functional groups on the synthesized hybrids; significantly broad peaks at 3414 and 3400 cm^−1^ were assigned to the secondary NH groups, appearing at 3077 and 3079 cm^−1^. The aliphatic (-C-H)str. appeared at 2974 and 2933 cm^−1^ for hybrids **9a** and **9b,** respectively. It was noteworthy to mention that there were distinctive (C = O) peaks at 1710 and 1705 cm^−1^ for **9a** and **9b**, respectively. In addition, the aromatic (C = C) stretching appeared at 1590 and 1588 cm^−1^; also, there were peaks at 1252 and 1258 cm^−1^, attributed to (>C = S) str. for **9a** and **9b,** respectively (for more details cf. experimental part). The analysis of the ^1^H-NMR spectra (cf. the experimental section) confirms the formation of the desired hybrid products through the presence of the aliphatic protons of the linkers, and the singlet at 4.18 ppm corresponding to 2 protons from the rhodanine ring in **9a** and the aliphatic protons from a conformationally flexible Piperazine side chain at the C-11 position of the **9b** in addition to the aromatic protons of the indoloquinoline ring system. Moreover, the ^1^H-NMR spectra revealed the presence of a peak at around 4.30 ppm corresponding to the aliphatic protons of the methyl group at N-5 of the quinoline ring. The ^13^C-NMR spectra showed a set of characteristic peaks at 201.60 and 205.10 ppm assigned to >C = S and at 170.90 and 177.5 ppm attributed to a >C = O peak for **9a** and **9b,** respectively. Hybrids **11a**, **11b**, **11c**, and **11d** showed characteristic FTIR peaks at 3419, 3413, 3410, and 3368 cm^−1^, assigned to the NH group, respectively. In addition, the (Ar-H)str. appeared at 3074, 3070, 3075 cm^−1^, and 3075 cm^−1^, and the aliphatic C-H stretching appeared at 2974, 2974, 2964 cm^−1^, and 2964 cm^−1^ for **11a**, **11b**, **11c**, and **11d**, respectively. Moreover, the >C = O absorption peak appeared at 1710, 1700, 1705, and 1702 cm^−1^ for **11a**, **11b**, and **11c**, respectively. The >C = S absorption band appeared at 1242, 1245, 1244, and 1250 cm^−1^ for **11a**, **11b**, **11c**, and **11d**, respectively. On the other hand, the exocyclic double bond (CH = C) at the C-5 position of rhodanine for all hybrids **11a**–**d** appeared in the range of 7.17–8.9 ppm as a singlet for the olefinic hydrogen, which is consistent with similarly reported analogs. In addition, the OH for **11b** displayed a broad singlet at 11.10 ppm while the NH signal appeared as a broad singlet at 9.04 and 9.10 ppm for **11a** and **11c,** respectively. The ^13^C-NMR showed, in addition to other ordinary peaks, characteristic signals at 196.10, 194.10, 202.60, and 198.8 ppm for >C = S signal while the peaks at 169.10, 168.6, 171.60, and 161.80 are characteristic for >C = O signals for **11a**, **11b**, **11c**, and **11d**, respectively. The structure assignment for **14** by IR and NMR showed characteristic peaks consistent with the corresponding structure with the active methylene proton appearing as a singlet at 4.09 ppm. This proves the installation of the rhodanine ring into the neocryptolepine core. In addition, the FTIR structure elucidates hybrid **16** with the appearance of broad bands at 3412 and 3420 cm^−1^, which are characteristic of the NH absorption band for **16a** and **16b,** respectively. Moreover, the phenolic OH signal of hybrid **16a** displayed a broad band at 3395 cm^−1^, while the IR of >C = O absorption for **16a** and **16b** appeared at 1710 cm^−1^. Furthermore, the >C = S thiocarbonyl stretching peak displayed at 1245 and 1249 cm^−1^ for **16a** and **16b**, respectively, while the ^1^H-NMR of **16a** and **16b,** respectively, showed singlets at δ = 8.90 and 8.84 ppm, which correspond to the exocyclic olefinic exocyclic double bond proton. On the other hand, an additional broad proton signal of OH appeared at 11.10 ppm for **16a**, while the NH broad singlet for **16b** displayed at 12.29 ppm. Furthermore, the mass spectra of the target hybrids are in conformity with the assigned structure and showed molecular ion peaks and fragmentation patterns corresponding to their molecular formula (for details cf. experimental section).

### 2.2. Cytotoxicity Screening

Seven compounds (Figure S1 supplementary Materials) were examined in vitro for their activities against MDA-MB-231 and HepG-2 using the MTT assay (Figures S2 and S3, supplementary file). The percentages of intact cells were calculated and compared to those of the control. The selection of the cancer cells was based on their prevalence especially in the develoPing countries [38,39]. All compounds suppressed the two cancer human cells in a dose-dependent manner using 5-fluorouracil (5-FU) as reference drug as shown in Figures S2 and S3 and Tables S1 and S2. In the case of MDA-MB-231 human breast carcinoma cells, the results are displayed in Table 1 and Table S3. Five compounds out of seven (**9a**, **11d**, **14**, **16a**, **16b**) were significantly potent against HepG-2 human liver cancer cells. The rest of the compounds had significantly less anticancer activities compared to that of 5-fluorouracil as reference standard, Table 1. The cytotoxicity of all new hybrids has already been studied on the BJ-1 human skin normal cell line, and the results show that these compounds have no significant cytotoxicity toward these healthy cells at the highest concentration used in this study.

#### 2.2.1. Cell-Cycle Analysis

After treating the HepG-2 cells with compounds (**9a**, **11c**, **5FU**) with concentrations 2 + 2998 µ, 2+ 2998 µ, and 0.78 + 2.999 µ, respectively, for 48h, the cells were stained with proPidium iodide and the cell-cycle assay was analyzed by using BD FACS Canto, Table S2. The results showed a notable difference between **9a**-, **11c**-, fluorouracil-treated groups, and the negative control. For **9a**-, **11c**-, and fluorouracil-treated groups, the cells were highly accumulated in the g0/g1 phase at 56.5% and 40.4%, respectively, slight decrease in S phase as well as in G2M phase when compared to control cells. The results showed cell-cycle arrest at the g0/g1 phase in the treated group.

For MDA cells, the results showed a notable difference between the **9a**-, **11c**-, fluorouracil-treated groups, and the negative control. For **9a**-, **11c**-, and fluorouracil-treated groups, the cells were highly accumulated in the g0/g1 phase at 61.4% and 1.4% respectively, with a slight decrease in the S phase at the same time as a slight decrease in the G2M phase in comparison with the control (untreated cells). The results showed cell-cycle arrest at the g0/g1 phase in the **9a**- and **11c**-treated groups while in the positive control group 98.8% were apoptotic cells; Figures S4–S19.

#### 2.2.2. Apoptosis Assay

A significant programmed cell-death was induced in the selected cancer cells by the investigated structures compared to control, Tables S1–S3. 9.59% apoptosis was detected in the case of MDA cells and 1.64% apoptosis in HEP-G2 cells. When cells were treated with **9a**, **11a** and 5-fluorouracil, we observed 1.43%, 2.92% and 18.5% viable cells and 89.6%, 51.2% and 46.9 % apoptotic cells, respectively in MDA cells among the tested compounds, **11a** showed the weakest apoptotic potential. Whereas, HEP-G2 cells demonstrated 27.6%, 11% and 2.82 % viable cells and 45.6%. 79.1% and 83.8% apoptotic cells, respectively. compound **9a** showed the weakest response.

### 2.3. Molecular Docking

#### 2.3.1. Analysis of 1t8i Crystal Structure

**Figure 3:**
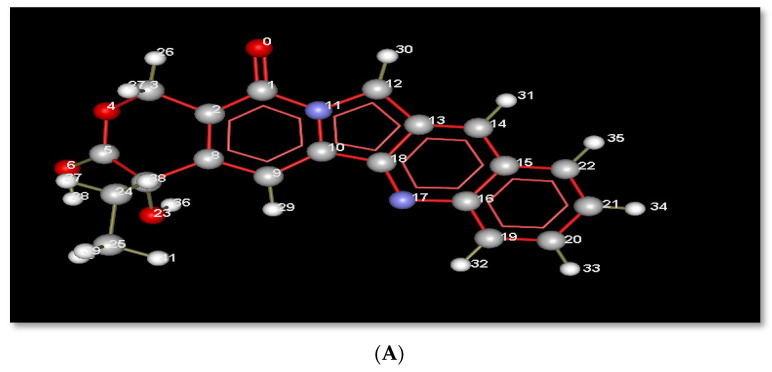

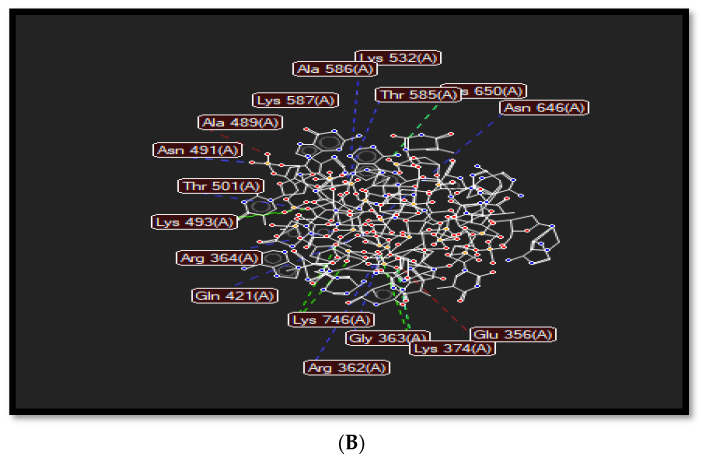
(**A**): Index number of acridine displayed in molecular interactions. (**B**) Representation of the molecular interactions of the 1t8i crystal structure, where blue dashed lines refer to hydrogen bond interactions, green dashed lines display electrostatic interactions and red lines show steric interactions.

The planar structure of acridine, as shown in Figure 3A, helps it to intercalate with DNA base pairs at the point of single-strand cleavage, as displayed in Figure 3. Acridine forms a hydrogen bond interaction with Arg364, as in Figure 3B, where that residue is responsible for drug resistance.

Various chemical interactions such as hydrogen bonds, electrostatic, and steric interactions occur to stabilize top1-DNA complexes and support the rational design of novel anticancer drugs. Hydrogen bonds are formed between several residues such as Arg362, Arg364, Asn491, Thr501, Lys532, Thr585, Lys587, and Asn646; electrostatic interactions are formed with His367, Lys374, Lys493, and Lys746; and steric interactions exist in Glu356, Arg488, Ala 489, and Asp533. These types of interactions force the residue’s side chain to adopt different conformations to provide a more stable complex, as dePicted in Figure 3B.

Hydrogen bonds (HBs) play different vital roles in physicochemical properties represented by length, strength, and spectroscoPic characteristics. Based on strength, three types of hydrogen bonds can be classified as weak (2–8 kcal mol^−1^), strong (10–20 kcal mol^−1^), and very strong (24–40 kcal mol^−1^). In a weak HB, a dipolar covalent bond is formed through proton attachment to a heteroatom while it is involved with another heteroatom by a weak electrostatic attraction; in a strong HB, the proton is bonded covalently to a heteroatom with an extra-long distance compared to a weak HB, while it is attached to another heteroatom by a short distance compared to a weak HB. These two heteroatoms are closer than van der Waals contact; in a very strong HB, the heteroatoms are much closer than a van der Waals contact, while the proton is almost placed equally between them [40].

Docked complex of topoisomerase I enzyme (red cartoons, gray segment of DNA, and red-blue DNA bases) with inhibitor **9a** (gray stick), Figure S20a,b, supplementary file.

Several interactions are represented in Figure S20. Pi or π-stacked interactions such as π–cation and anion are related to the conformational stability of potential drugs [41]. For chain A, there are π—cation and anion interactions between inhibitor **9a** with residue Arg364 (5.80 Aᵒ, 7.05Aᵒ) and with the DNA strand. A *Pi*–sigma interaction (*Pi*–Sulphur with His632, 4.23 Aᵒ) leads to a charge transfer that enforces drug intercalation in the receptor-binding site [41].

Van der Waals interactions created strong, firm surroundings with protein residues and DNA bases, thereby forming a stable protein-inhibitor complex between residues Lys532, Ile535, Asn631, Gln633, and Asn722; conventional hydrogen bonds with Arg488, Asp533, and Thr718; and carbon–hydrogen bonds with DNA bases and compound **9a** (Figure S20).

In the current study, conventional hydrogen bonds were formed with DNA bases. Moreover, van der Waals interactions were formed between inhibitor **9b** and residues Arg488, Lys532, Gly717, Thr718, Leu721, and Asn722 and with DNA strands, besides Pi-alkyl with His 632 (Figure S21b). These types of interactions also exist in **11c** (Figure S22b).

Lone-pair–π (lp–π) is considered a non-covalent bond interaction proposed to form more stable DNA and protein structures, separately or in complexes [41]. Here *Pi*–lone pair interactions occurred between the phenol ring of inhibitor 4 and residue Asn 722, which stabilizes the topoisomerase-I and inhibitor **11d** complexes, Figure S23b.

*Pi*–alkyl interactions lead to more stable protein–ligand complexes [41]. A Pi–alkyl interaction was formed between residue Leu721 and two rings of inhibitor **11d**; one is a phenol ring while the second is the 2-thioxothiazolidin-4-one ring of compound **11d** (Figure S23b). The rest of the chemical compounds showed different types of interactions, such as compounds **14** and **16a**–**b** (Figures S24–S26b).

#### 2.3.2. Molecular Hydrophobic Potential Analysis

The seven compounds (Figure S1) in the current study show good docking scores from −5.90 to −7.77 kcal/mol, for compounds **11d** and **16b**, respectively (Table S4), which can be demonstrated through many parameters such as H-bonds (Tables S5 and S6), lipophilic match surface (SLL), hydrophilic match surface (SHH), ligand buried surface area (Sburied), total surface area (Stotal), the fraction of matching total surface (Match1), the fraction of matching hydrophobic surface (Match2), and stacking (Stack.) (Stack.Gua-π), All the above-mentioned parameters are listed in Table S6, supplementary file.

In Table S6, supplementary file, all seven inhibitors show Match2 in the range of 0.69–0.84, so the hydrophobic interactions are the main forces that stabilize these compounds, while hydrogen bonds representing hydrophilic interactions are few.

All seven docked compounds are depicted in Figures S20–S26, which define the inhibition mechanism of topoisomerase I and their inhibitor complexes (Table S4). Compounds **16b** and **9a** are the most active compounds with a binding energy of −7.77 and −7.51 Kcal/mol. The less active compound was compound **11d** (−5.9 Kcal/mol); the decrease in binding energy was due to increasing the size of the inhibitors, which will not fit the binding cavity, as listed in the volume properties (Table S5), although the hydrophobicity (Match2) was high for less-active compound **11d** (0.8127) due to the more aromatic rings in such a compound when compared to compound **9a** (0.7296) and **16b** (0.6941); Table S6, supplementary file.

The bioactivity scores were good for compounds **9a**, **9b**, **14**, **16a**, and **16b,** respectively, while the other two compounds, **11c** and **11d**, were less active (Table S7, supplementary file). For that reason, compound **9a** (as a highly bioactive compound among the seven compounds) and **11c** (as the least active compound) were selected for cell-cycle analysis.

#### 2.3.3. Insilico Pharmacokinetics Evaluation and Drug Likeness

*Insilico* assessment of pharmacokinetic properties and drug likeness was performed on the biologically tested compounds **9a**, **9b**, **11c**, **11d**, **14**, **16a**, **16b** using Swiss ADME [42] and Molsoft software. When compounds demonstrate positive value, they are regarded as promising drug-like scaffold as reported [43]. In the table below, Table 2, six compounds **9a**, **9b**, **11c**, **11d**, **14**, **16a** out of seven expressed positive results between 0.32 and 1.26, whereas the best score was compound **9b** (1.26). According to LiPinski’s rule of five, five compounds **9a**, **11c**, **14**, **16a**, **16b** are considered as drug-like without rule violations having good bioavailability.

Five of the tested compounds **9a**, **9b**, **14**, **16a**, **16b** had log *P* values not exceeding 5, which demonstrates promising tolerability with cell membranes, this comes consistent with their bioactive score recorded in the molecular docking. In addition, the topological polar surface area ranges from 107.55 to 135.79. Hydrogen bond acceptors are between 2 and 5 acceptors while the donors are either 1 or 2 following LiPinski’s rule. As referenced [44,45], the blood brain barrier (BBB) score should range are between 0 and 6. The tested structures showed a minimum score of 2.16 and maximum one of 3.64, Table 2

## 3. Materials and Methods

### 3.1. Chemistry

Chemical reagents and solvents were purchased from commercial stores; solvents were prepared according to standard protocols. Each chemical product was identified by Thin Layer Chromatography (TLC) from kieselgel F254 precoated plates, Merck. Melting points were measured using a Thomas–Hoover caPillary apparatus. FT-IR spectra were verified and stored on films by KBr plates using a Nicolet 550 Series II Magna FT-IR spectrometer. ^1^H NMR and ^13^C NMR spectra were identified by a Bruker Avance (400 MHz, 100 MHz respectively) spectrophotometer in the main chemical warfare laboratories, Egyptian DMSO-d_6_ with TMS as the internal standard, where J (coupling constant) values were estimated in Hertz (Hz) and chemical shifts were recorded as parts per million (ppm) on the δ scale. Mass spectra (MS) were recorded on a thermos scientific trace 1310 gas chromatograph at the Fungi National Centre, Al-Azhar University, Egypt. Moreover, intermediates **3**, **4**, and **5** were prepared previously by reported procedures [15,16,19,22]. Anticancer activity was carried out at the National Research Center. Flow cytometry measurements were performed at the Center of Excellence in Cancer Research (CECR), Tanta University, Egypt.

### 3.2. General Procedure for the Synthesis of 11-Aminoalkylene Amino Neocryptolepine Derivatives ***7a,b***

11-Chloroindoloquinoline **5** (0.1 mmol) and an excess of the appropriate amino alkylene amine **6 a**,**b** (40 eq.) were heated together at 135−155 °C for 5–10 10 min to give crude oil with brown color. Purification was done by flash chromatography using AcOEt−2N ammonia in MeOH (9:1) affording pure light orange crystals.

*N1-(5-methyl-5H-indolo[2,3-b]quinolin-11-yl)ethane-1,2-diamine***7a**. Yellowish-orange solids; yield: 76%; M.p: 108–110 °C; FT-IR (KBr) cm^−1^: 3242, 2859, 1615, 1556, 1446, 1446, 1417, 1391, 1337, 1323 and 1275 cm^−1^; ^1^H-NMR (400 MHz; CD_2_Cl_2_) δ: 8.10 (d, *J* = 2.2 Hz, 1H), 8.08–8.05 (m, 1H), 7.77–7.74 (m, 1H), 7.60 (dd, *J* = 9.1, 2.3 Hz, 1H), 7.53 (d, *J* = 9.1 Hz, 1H), 7.45 (ddd, *J* = 8.1, 7.2, 1.0 Hz, 1H), 7.22–7.18 (m, 1H), 6.35 (s, 1H), 4.18 (s, 3H), 3.74 (q, *J* = 5.2 Hz, 2H), 2.98–2.95 (m, 2H), 1.51 (s, 2H); MS (EI), *m/z*: Calcd: 290.36 (C_18_H_17_ClN_4_), found: [M]^+^ (290.32).

*N-(3-(4-(3-aminopropyl)Piperazin-1-yl)propyl)-5-methyl-5H-indolo[2,3-b]quinolin-11-amine* **7b**: Yellow solid; yield: 82%; M.p: 135 °C; ^1^HNMR(400 MHz; CD_2_Cl_2_): δ 8.22 (dd, *J* = 8.3, 1.1, 1H), 7.93 (d, *J* = 7.7, 1H), 7.74–7.72 (m, 1H), 7.70 (dd, *J* = 6.7, 1.3, 1H), 7.66 (dd, *J* = 8.6, 1.2, 1H), 7.39 (ddd, *J* = 8.0, 7.2, 1.0, 1H), 7.34 (ddd, *J* = 8.2, 6.8, 1.4, 1H), 7.16–7.12 (m, 1H), 7.05 (t, *J* = 5.0, 1H), 4.24 (s, 3H), 3.99 (q, *J* = 5.0, 2H), 2.76 (t, *J* = 6.8, 2H), 2.65 (t, *J* = 5.0, 2H), 2.60–2.59 (m, 2H), 2.46 (t, *J* = 7.4, 2H), 1.92 (quint, *J* = 5.0, 2H), 1.70–1.63 (m, 10H).^13^C NMR (100 MHz; DMSO): δ 157.1, 152.8, 148.6, 137.9, 130.3, 125.4, 124.2, 121.7, 120.5, 118.6, 117.2, 116.2, 114.6, 106.7, 58.4, 56.7, 53.8, 53.1, 49.4, 40.8, 32.7, 30.4, 26.3. MS (EI), *m/z*: Calcd: 431.29 (C_26_H_34_N_6_), found: [M]^+^ (431.29).

### 3.3. Synthesis of Neocryptolepine–rhodanine Hybrids ***9a,b***

A mixture of **7a**,**b** (1 mmol), carbon disulfide (1 mmol), and ethyl bromoacetate **8** (2.01 mmol) were stirred in acetonitrile (1.5 mL) at room temperature. The excess solventwas evaporated under reduced pressure. The result solid was purified using column chromatography (60-120 mesh), eluent used was methanol–Chloroform in ratio 1:9.

*3-(2-((5-methyl-5H-indolo[2,3-b]quinolin-11-yl)amino)ethyl)-2-thioxothiazolidin-4-one* **9a**: Red solid; *yield*: 76%, M.p: 240–243 °C; FT-IR (KBr) cm^−1^ 3414, 3077, 2974, 1710,1604, 1590, 1252, 1168 and 743; ^1^H NMR (DMSO-d*_6_* 400 MHz):4.11 (t, 2H, CH_2_), 4.13 (t,2H,CH_2_), 4.18 (s, 2H, CH_2_), 4.35 (s, 3H, N-CH_3_), 7.49–8.08 (m, 8H, Ar-H), 9.04 (br.s, 1H, NH); ^13^C NMR (100 MHz, DMSO-*d_6_*): δ 201.6, 170.9, 135.4, 129.3, 128.9, 126.4, 115.8, 53.1, 39.7, 38.6, 31.1. MS (EI), *m*/*z*: Calcd: 406.52 (C_21_H_14_N_4_OS_2_), found: [M]^+^ (406.09).

*3-(3-(4-(3-((5-methyl-5H-indolo[2,3-b]quinolin-11-yl)amino)propyl)Piperazin-1-yl)propyl)-2-thioxothiazolidin-4-one* **9b**: Red solid; yield: 80%, M.p: 184–187 °C; FT-IR (KBr) cm^−1^ 3400, 3079, 2933, 1705, 1588, 1258, 1178 and 748; ^1^H NMR (DMSO-d_6_ 400 MHz): 1.24 (m, 4H, CH_2_), 1.89 (m, 4H, CH_2_), 2.52 (t, 8H, CH_2_), 3.62 (t, 2H, CH_2_), 3.71(q, 2H, CH_2_), 4.23 (s, 2H, CH_2_), 4.26 (s, 3H, N-CH_3_), 7.17–7.93 (m, 8H, Ar-H); ^13^C NMR (100 MHz, DMSO-*d_6_*): δ 205.1, 177.5, 145.1, 128.7, 127.3, 113.1, 53.1, 39.7, 38.6, 31.6, 27.7, 23.6. MS (EI), *m*/*z*: Calcd: 546.75 (C_29_H_34_N_6_OS_2_), found: [M]^+^ (546.22).

### 3.4. Representative General Procedure for the Synthesis of Compounds ***11a***–***d***

#### 3.4.1. Pathway A

A mixture of **9a**,**b** (1.37 mmol), appropriate aldehyde **10 a,b** (1.39 mmol) and ammonium acetate (2.05 mmol) was stirred in acetic acid and refluxed. After reaction completioin, acetic acid was removed under vacuum. The preciPitated residue was washed, extracted by dichloromethane, dried over magnesium sulfate then filtrated and evaporated under vacuum to afford compounds **11a**–**d**.

#### 3.4.2. Pathway B: One-Pot Reaction Procedure for the Synthesis of **11a**–**d**

To a mixture of amines **7a**,**b** (1 mmol) and CS_2_ (2 mmol) in (2 mL) DMF, ethyl chloroacetate **12** (1 mmol) was added dropwise under stirring at room temperature for 30 min–1 h, then 3 mmol of KOH and 1 mmol of aromatic aldehydes **10 a**,**b** were added. The resulting mixture was stirred at room temperature overnight, then poured into crushed ice, and the preciPitate formed was washed three times with water, dried, and finally recrystallized from ethanol to afford pure **11a**–**d** in good yields.

*(Z)-5-benzylidene-3-(2-((5-methyl-5H-indolo[2,3-b]quinolin-11-yl)amino)ethyl)-2-thioxothiazolidin-4-one* ***11a***: Brown solid; yield: 78%, mp:150–153 °C; FT-IR (KBr): cm^−1^ 3419, 3074, 2974, 1710, 1604, 1588, 1242, 1168 and 743., ^1^H NMR (DMSO-d_6_ 400 MHz): 1.20 (t, 2H, CH_2_), 3.79 (t, *2H*, CH_2_), 4.35 (s, 3H, N-CH_3_), 7.22–8.61(m, 13H, Ar-H), 7.79 (br.s, 1H, active RH), 9.04 (br.s, 1H, NH); ^13^C NMR (100 MHz; DMSO): δ 196.1, 169.1, 146.7, 144.2, 139.2, 129.2, 127.2, 118.8, 55.3, 39.8, 33.11. MS (EI), *m*/*z*: Calcd: 494.12 (C_28_H_22_N_4_OS_2_), found: [M]^+^ (493.8).

*(Z)-5-(4-hydroxybenzylidene)-3-(2-((5-methyl-5H-indolo[2,3-b]quinolin-11-yl)amino)ethyl)-2-thioxothiazolidin-4-one* **11b**: Brown solid; yield: 70%, mp:162–165 °C; FT-IR (KBr): cm^−1^ 3413, 3390, 3070, 2974, 1700, 1604, 1598, 1245, 1168 and 745; ^1^H NMR (DMSO-d_6_ 400 *MHz*): 3.20 (t, 2H, CH_2_), 3.79 (t, 2H, CH_2_), 4.36 (s, 3H, N-CH_3_), 7.28–8.61(m, 13H, Ar-H), 7.79 (br.s, 1H, CH), 9.06 (br.s, 1H, NH), 9.76 (br.s, 1H, OH); ^13^C NMR (100 MHz; DMSO): δ 194.1, 168.6, 146.7, 143.2, 137.2, 129.2, 128.2, 114.8, 51.3, 37.5, 31.6. MS (EI), *m*/*z*: Calcd: 510.12 (C_28_H_23_N_4_OS_2_), found: [M]^+^ (509.8).

(*Z*)-5-benzylidene-3-(3-(4-(3-((5-methyl-5*H*-indolo[2,3-b]quinolin-11-yl)amino)propyl)Piperazin-1-yl)propyl)-2-thioxothiazolidin-4-one **11c**: Brown solid; yield: 78%, mp: 174–176 °C; FT-IR (KBr): cm^−1^ 3410, 3075, 2964, 1705, 1612, 1590, 1244, 1200 and 740. ^1^H NMR (DMSO-d_6_ 400 MHz): 1.61 (m, 2H, CH_2_), 1.81 (m, 2H, CH_2_), 2.20 (t, 8H, CH_2_), 3.09 (t, 2H, CH_2_), 3.40 (q, *2H*, CH_2_), 3.81(s, 2H, CH_2_), 3.87(s, 2H, CH_2_), 4.26 (s, 3H, N-CH_3_), 7.05–8.46 (m, 13H, Ar-H), 7.17 (br.s, 1H, CH), 9.10 (br.s, 1H, NH); ^13^C NMR (100 MHz; DMSO): δ 202.6, 171.6, 146.7, 141.2, 133.2, 127.2, 117.8, 51.3, 41.3. 41.8, 37.5, 31.2, 23.6. MS (EI), *m*/*z*: Calcd: 634.86 (C_36_H_38_N_6_OS_2_), found: [M]+ (634.22).

(*Z*)-5-(4-hydroxybenzylidene)-3-(3-(4-(3-((5-methyl-5*H*-indolo[2,3-b]quinolin-11-yl)amino)propyl)Piperazin-1-yl)propyl)-2-thioxothiazolidin-4-one **11d**: Brown solid; yield: 69%, mp: 90–93 °C; IR (KBr): cm^−1^ ν 1 3368, 3395, 3075, 2964, 1702, 1612, 1598, 1250, 1189 and 742. ^1^H NMR (DMSO-d_6_ 400 MHz): 1.15 (m, 2H, CH_2_), 1.85 (m, 2H, CH_2_), 2.25 (t, 8H, CH_2_), 3.88 (t, 2H, CH_2_), 3.98 (q, 2H, CH_2_), 4.08 (s, 2H, CH_2_), 4.14 (s, 3H, N-CH_3_), 7.29–8.57 (m, 13H, Ar-H), 8.12 (br.s, 1H, CH), 9.76 (br.s, 1H, OH).; ^13^C NMR (100 MHz; DMSO): δ 198.8, 161.8, 140.7, 141.2, 135.2, 128.6, 114.8, 51.3, 41.3. 41.9, 37.5, 31.6, 22.8. MS (EI), *m*/*z*: Calcd: 650.86 (C_36_H_39_N_6_O_2_S_2_), found: [M]^+^ (650.22).

## 4. Synthesis of 11-Amino Rhodanine Neocryptolepine Hybrid 14

A mixture of **5** (1 mmol) was dissolved in dry DMF (10 mL). Triethylamine (TEA) (5 mmol) and 3-aminorhodanine 13 (1 mmol) were refluxed while waiting for the initial materials to disappear as observed by TLC (4 h). The reaction mixture was cooled and poured into ice water, then extracted from chloroform, dried over anhydrous sodium sulfate, then filtered and evaporated to give pure hybrid **14**.

3-((5-*methyl*-5H-indolo[2,3-b]quinolin-11-yl)amino)-4-thioxothiazolidin-2-one **14**. Yellow solid; *yield*: 84%; m.p: 210–214. 1H NMR (DMSO-d_6_ 400 MHz) ppm: 4.08 (s, 3H, N-CH_3_), 4.09 (m, 2H, CH_2_), 7.28–7.89 (m, 8H, Ar-H) 12.4 (br.s, 1H, NH).^13^C NMR (100 MHz; DMSO): δ 196.1, 183.07, 155.3, 148.6, 146.4, 143.2, 140.7, 129.5, 128.4, 127.1, 117.50, 107.1, 97.8, 48.4, 35.1. (EI-MS), *m*/*z* (C_19_H_14_OS_2_N_4_) calcd., 378.47[M]^+^; found, 378.06.

## 5. General Procedure for the Synthesis of **16a,b**

Compound **14** (1 mmol) was reacted with appropriate aldehydes **15a**,**b** (1 mmol) in the presence of sodium acetate (1.2 mmol) in glacial acetic acid (3 mL) under reflux with stirring for 6 to 8h. After the completion of the reaction, the acetic acid was removed under vacuum. The residue was washed with water, then extracted by dichloromethane, dried over magnesium sulfate, filtrated, and evaporated under vacuum to give the desired products **16a**,**b**.

*(Z)-5-(4-fluorobenzylidene)-3-((5-methyl-5H-indolo[2,3-b]quinolin-11-yl)amino)-4-thioxothiazolidin-2-one* **16a**: Brown solid; yield: 80%; m.p: 171–173 °C. FT-IR (KBr) cm^−1^: 3410 (NH), 3077 (=CH sym), 2974 (C-H sym), 1710 (C = O), 1604(C = N), 1242 (C = S), 1168 (C-N), 748 (C-S), ^1^H NMR (DMSO-d6 400 MHz): 4.10 (s, 3H, N-CH_3_), 7.19–7.69 (m, 12H, Ar-H), 7.7 (s, 1H, CH). (EI-MS), *m*/*z*: (C_26_H_17_FN_4_OS_2_) calcd, 484.57 [M]^+^; found, 484.09.

*(Z)-3-((5-methyl-5H-indolo[2,3-b]quinolin-11-yl)amino)-5-(thiophen-2-ylmethylene)-4-thioxothiazolidin-2-one* **16b**. Brown solid; yield: 78%, m.p: 169–172 °C. FT-IR (KBr) cm^−1^: 3420 (NH), 3078 (=CH sym), 2973 (-CH sym), 1710 (C = O), 1604 (C = N), 1598 (C = C, Ar), 1249 (C = S), 1168 (C-N), 744 (C-S), ^1^H NMR (DMSO- d_6_, 400 MHz): 4.10 (s, 3H, N-CH_3_), 7.28–7.91(m, 11H, Ar-H), 8.84 (s, 1H, CH), 12.29 (s, 1H, NH). EI-MS, *m*/*z* (C_24_H_16_N_4_OS_3_) calcd. 472.61 [M]^+^; found, 462.05.

All the compounds are dePicted in Figure S1. See supplementary file.

In vitro antiproliferative bioassay, Materials and Methods, cell cultures:

The cell lines of M.D. Anderson (MDA) and metastasized breast cancer (MB) to form MDA-MB-231, along with human hepatoma (HepG-2) and cell culture materials, were purchased from the American Type Culture Collection (Rockville, MD) and maintained in Dulbecco’s Modified Eagle Medium (DMEM), which was supplemented with 10% heat-inactivated FBS (fetal bovine serum), 100 U/mL penicillin, and 100 U/mL streptomycin. The cells were grown at 37 °C in a humidified atmosphere of 5% carbon dioxide (CO_2_).

Evaluation of cell proliferation by MTT assay:

The cytotoxicity activities of the substances under research were assessed using human cancer cell lines MDA-MB-231 and HepG-2, in which viable cells mitochondrial dehydrogenases was used to reduce [3-(4,5-dimethyl-2-thiazolyl)-2,5-diphenyl-2H-tetrazolium bromide [46,47].

Prior to the MTT assay, and in a serum free medium, cells were dispensed in a 96 well sterile microplate (5 × 104 cells/well). Media were removed with cautious upon incubation and 40 µL of MTT (2.5 mg/mL) were added to each well. Incubation was then done at 37 °C for additional 4 h with serial concentrations of each tested compound or reference standard (doxorubicin and 5-flourouracil). All compounds and references were dissolved in DMSO, for 48 h. 200 µL of DMSO was added to solubilized purple formazan dye crystals. Spectra Max Paradigm Multi-Mode microplate reader was used to measure absorbance at 570 nm. The mean percentage of viable cells compared to the untreated control cells reflected the relative cell viability. All procedures were performed as triplicate and repeated on three different days, values were recorded as mean ± SD and IC_50s_ were calculated using SPSS and probit analysis (IBM Corp., Armonk, NY, USA).

### 5.1. Molecular Docking

The current class of compounds (Figure S1) is structurally related to camptothecin and the prototyPical examples of two groups of top1 toxins, namely, indenoisoquinolines and indolocarbazePine [48]. All structurally distinct chemicals can intercalate between DNA base pairs at the location of single-strand cleavage because of their planar structures. Some chemicals include a free electron pair close to Arg364, a residue that, if altered, gives resistance to such medications (**9a**, Figure S20b; **16a**, Figure S25b; **16b**, Figure S26b. Supplementary Materials). In addition to the predicted intercalative binding mode, chemotype-specific interactions with Asn352 (**11c**, Figure S22b) cause the residue to adopt a different side chain conformation to bind the chemicals. To help rationally design whole new structural classes of innovative neocryptolepine–rhodanine hybrids, previous X-ray structures for camptothecin, indenoisoquinolines, and indolocarbazePine have been released and proved that compounds with vastly diverse properties can stabilize top1-DNA covalent complexes [49].

**Figure 4:**
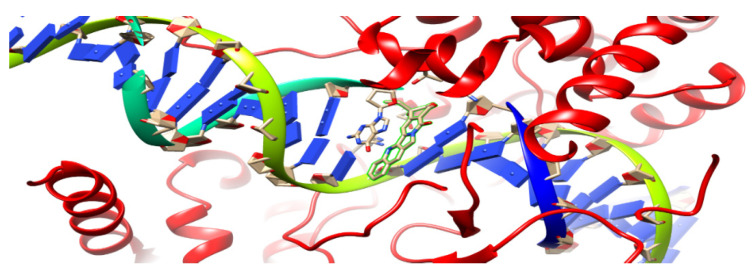
Ribbon diagram of 1t8i crystal structure; the highlighted part demonstrates acridine connected to a single DNA strand.

In the current research, a 1t8i crystal structure [49] with a resolution of 3 Aᵒ was used as the target receptor for the present docking study; Figure 4. Autodock 4 was used for the docking study because it is more accurate with crystal structures that bind to nucleic acids [50]. It was the first docking platform used to shape the conformational flexibility of a ligand. It consists of two packages, which are AutoGrid and AutoDock, where AutoGrid calculates the interaction noncovalent energy between receptor and prob atoms when put on various grid points of the lattice. AutoGrid produces electrostatic and desolvation maps that are used by Autodock to control the docking process of ligands.

The Lamarckian genetic algorithm (LGA) [51] was used by AutoDock 4.2.6 to create molecular conformations of the selected compound. One hundred docking runs were executed, with a population of 150 random individuals and a maximum number of 2,500,000 energy evaluations. The grid produced automatically contains the selected docking site of the protein structure.

Raccoon is a graphical interface for preparing AutoDock virtual screenings [52]. Chimera [53] and Molegro Molecular Viewer [54] are packages used to visualize the 1t8i crystal structure and its binding mode. BIOVIA Discovery Studio v21.1.1.0.20298 [55] was used to illustrate enzyme–inhibitor interactions.

### 5.2. Molecular Hydrophobic Potential Analysis

Hydrophobicity is a characteristic that determines the strength of a receptor–ligand complex, as mentioned in [56], while hydrogen bonds are the main interactions inside living species with a range from 0 to 1 as a representation of perfect geometry. SLL, area of lipophilic match surface (Aᵒ); SHH, area of hydrophilic match surface (Aᵒ); Sburied, area of ligand buried surface which equals SLL+ SLH + SHL +SHH (Aᵒ); Stotal, the fraction of matching total surface, (SLL + SHH)/(Stotal); Match1, the fraction of matching hydrophobic surface, 2SLL/(2SLL + SHL + SLH + SLH); Match2, scored in a range from zero to one, where zero refers to hydrophilic and one refers to hydrophobic; Stack, the stacking process that occurs between aromatic groups where they arranged in parallel; Stack.Gua-π, guanidinium groups [57], which exist in arginine residue, have a flat shape, a positive charge, and stack with aromatic contacts, making for better interactions.

## 6. Conclusions

A series of novel neocryptolepine–rhodanine hybrids have been synthesized and evaluated against hepatocellular carcinoma (HepG-2) and human breast (MDA-MB-231) cancer cell lines. Most of the synthesized hybrids exhibited potent cytotoxicity on human HepG-2 cancer cells and were similarly as potent as the standard drug 5-fluorouracil (5-FU). In addition, the tested hybrids exhibited high selectivity toward cancer cells rather than the normal skin human cell line (BJ-1) at the highest concentration used in this study. Molecular docking studies revealed that the presence of planar indoloquinoline fusing four rings and flexible side chain groups together improves DNA intercalation and the inhibition of DNA topoisomerase activity. Furthermore, in silico assessment for pharmacokinetic properties was performed using *Swiss* ADME on the most potent compounds. Compounds **9a**, **11c**, **14**, **16a**, and **16b** showed good bioavailability, with no LiPinski’s rule violations. Compounds **9a**, **9b**, **14**, **16a**, and **16b** reflected high tolerability with cell membranes with their log*P* values. In addition, drug-likeness scores were recorded using Molsoft (available from http://www.molsoft.com/mprop/) besides Molinspiration (available from https://www.molinspiration, and compounds) **9a**, **9b**, **11c**, **11d**, **14**, and **16a** expressed positive drug-likeness values ranging from 0.32 to 1.26. The best score (1.26) was recorded by compound **9b**. Further variations in substituents and substitution patterns are currently underway to obtain more potent analogs showing in vitro and in vivo activities.

## Figures and Tables

**Table 1:** IC_50_ of the examined compounds on two different cancer types.

Compound Code	IC_50_ (µM) ± SD	
	MDA-MB-231	HepG-2
**9a**	37.7 ± 3.9	27.7± 3.8
**9b**	30.0 ± 3.8	36.1 ± 4.2
**11c**	22.7 ± 3.1	35.6 ± 4.1
**11d**	25.3 ± 3.1	25.4± 3.3
**14**	29.9 ± 3.6	23.6 ± 3.8
**16a**	38.8 ± 4.3	24.6± 3.3
**16b**	31.9 ± 3.8	25.6 ± 3.1
5-Fluorouracil	12.0 ± 2.5	28.0 ± 2.3

**Table 2:** Results of the in silico pharmacokinetics assessment of the tested compounds.

Molecule	M.Wt	HBA	HBD	MR	TPSA	GI absorption	BBB	iLogP	Bioavailability	Drug Likeness
**9a**	406.52	2	1	124.82	107.55	High	3.64	2.98	0.55	0.41
**9b**	546.75	4	1	174.78	114.03	High	3.62	4.49	0.55	1.26
**11c**	406.52	2	1	124.82	107.55	High	2.99	5.62	0.55	1.03
**11d**	650.86	5	2	206.42	134.26	Low	2.16	5.05	0.55	1.07
**14**	378.47	2	1	115.21	107.55	High	3.74	2.64	0.55	0.22
**16a**	484.57	3	1	144.78	107.55	Low	3.34	4.07	0.55	0.32
**16b**	472.61	2	1	142.69	135.79	Low	3.39	3.76	0.55	−0.02

## Data Availability

Available from the corresponding author on reasonable request.

## Supplementary Materials

The following supporting information can be downloaded at: https://www.mdpi.com/article/10.3390/molecules27217599/s1, Figure S1: Seven active neocryptolepine analogs. Figures S2 and S3: Dose-dependent anticancer activities of 7 compounds on MDA-MB-231 and HepG-2 cancer cell lines. Figures S4–S11: Flow cytometry analysis of positive control, negative control, and compounds **9a** and **11c** in the case of HepG-2. Figures S12–S15: Apoptosis analysis of positive control, negative control, and compounds **9a** and **11c** in the case of HepG-2. Figures S16–S19: Apoptosis analysis of positive control, negative control, and compounds **9a** and **11c** in the case of MDA. Figure S20–26: Docked complex of topoisomerase I enzyme and compounds **9a**, **9b**, **11c**, **11d**, **14**, **16a,** and **16b**. Table S1: Percentage of cell growth inhibition of tested compounds with different concentrations in the case of the MDA-MB-231 cell line. Table S2: Percentage of cell growth inhibition of tested compounds with different concentrations in the case of the HepG-2 cell line. Table S3: IC_50_ of the compounds against the two cancer types according to the MTT assay. Tables S4 and S5: Concentrations in the case of HepG-2 and MDA. Table S6: Results of cell-cycle and apoptosis analysis. Table S7: Inhibition mechanism of topoisomerase I seven inhibitor complexes. Table S8: Calculations of physical properties for the seven compounds. Table S9: Hydrophobic interactions of the seven compounds. Table S10: Calculation of bioactivity scores for compounds **9a**–**16b**.
