# Supplementary material for: Design, Synthesis, and Antiproliferative Activity of Novel Neocryptolepine–Rhodanine Hybrids
Source: Molecules. 2022 Nov 5;27(21):7599. doi: 10.3390/molecules27217599 (PMC9656124; doi:10.3390/molecules27217599)
Supplement: Supplementary file 1 [file molecules-27-07599-s001.zip › molecules-1912050-supplementary.pdf]

Design, Synthesis, and Antiproliferative Activity of Novel NeocryptolePine–Rhodanine Hybrids

Mohamed El-Bahnsawye [1], Mona K. Abo Hussein [2], Elshaymaa I. Elmongy [3], Hanem Mohamed Awad [4], Aliaa Abd El-Kader Tolan [5], Yasmine Shafik Moemen [5], Ahmed El-Shaarawy [5] and Ibrahim El-Tantawy El-Sayed [1,*]

[1] Chemistry Department, Faculty of Science, Menoufia University, Shebin El-Kom 32511, Egypt
[2] Clinical Microbiology and Immunology Department, National Liver Institute, Menoufia University, Shebin El-Kom 32511, Egypt
[3] Department of Pharmaceutical Sciences, College of Pharmacy, Princess Nourah Bint Abdulrahman University, P.O. Box 84428, Riyadh 11671, Saudi Arabia
[4] Tanning Materials and Leather Technology Department, National Research Centre, Dokki, Giza 12622, Egypt
[5] Clinical Pathology Department, National Liver Institute, Menoufia University, Shebin El-Kom 32511, Egypt
* Correspondence: ibrahimtantawy@yahoo.co.uk

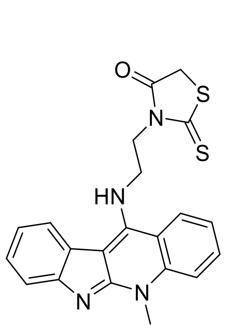

9a

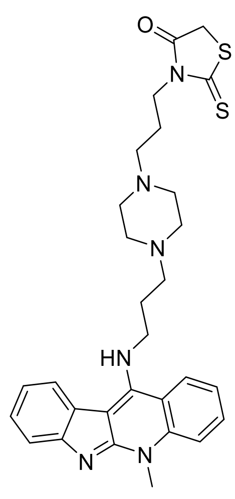

9b

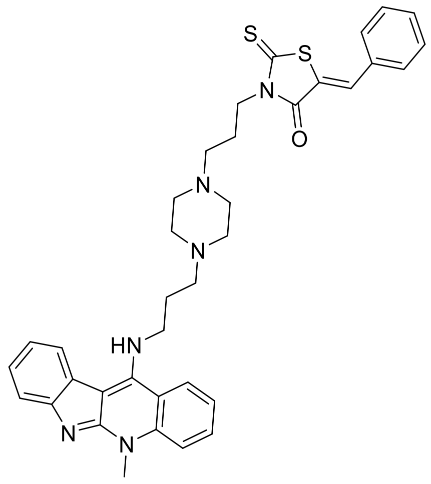

11c

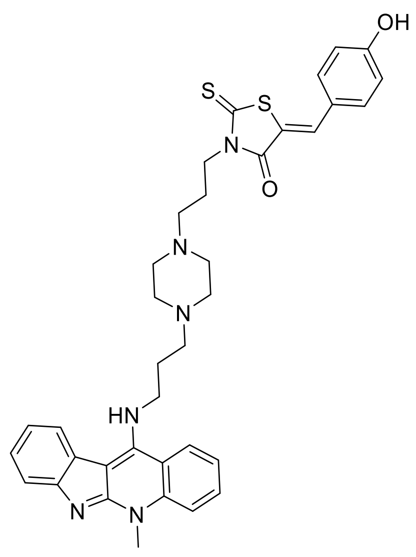

11d

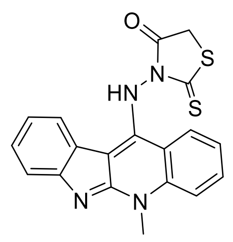

13

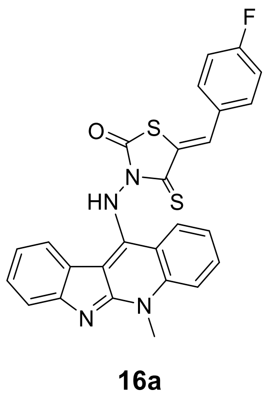

16a

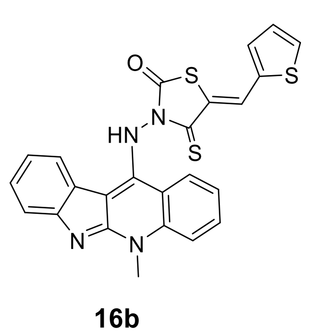

16b

Figure S1: Seven active neocryptolepine analogues.

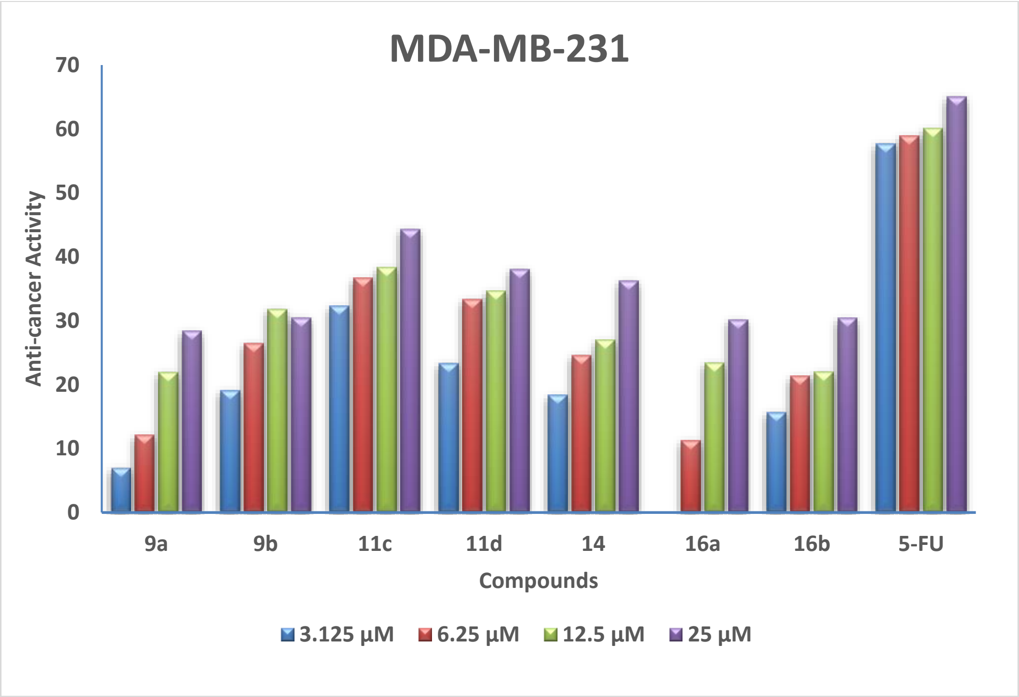

Figure S2: Dose dependent anticancer activities of 7 compounds on MDA-MB-231 cancer cell line.

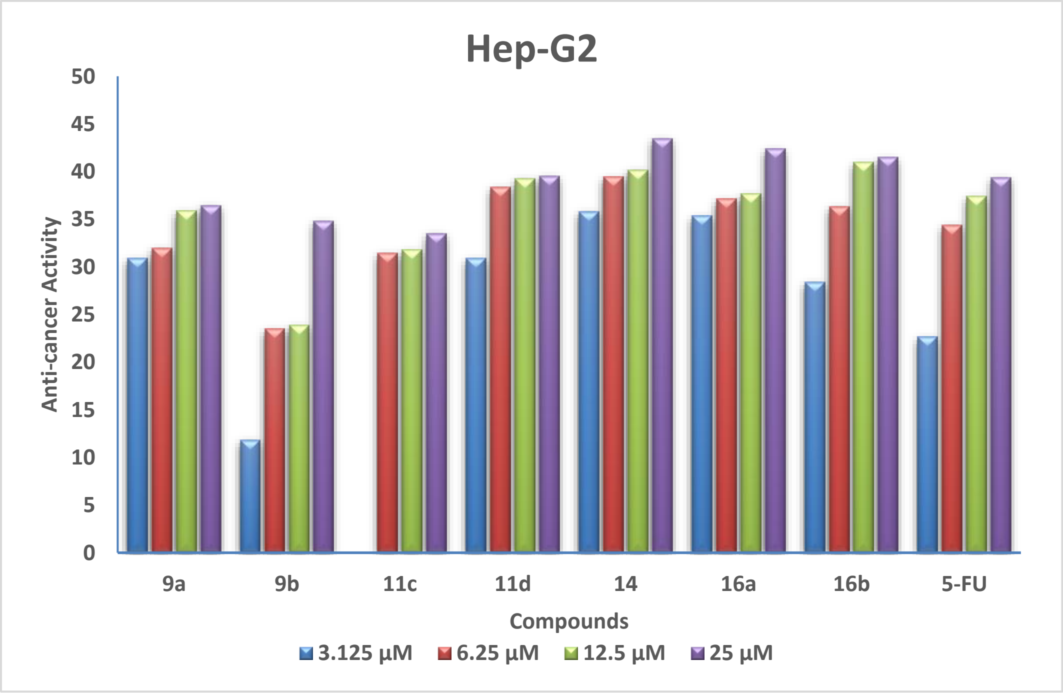

Figure S3: Dose dependent cytotoxic activities of 7compounds on Hep-G2 cancer cell line.

Table S1: % of cell growth inhibition of tested compounds with different concentrations in case of MDA-MB-231 cancer cell line.

Code	3.125 µM	6.25 µM	12.5 µM	25 µM
**9a**	7.02095	12.12059	21.93153	28.38017
**9b**	19.08022	26.46909	31.78334	30.465
**11c**	32.28411	36.68881	38.33418	44.33316
**11d**	23.33163	33.35718	34.65508	38.04803
**14**	18.38528	24.59888	26.96985	36.25958
**16a**	0.030659	11.25192	23.41339	30.15841
**16b**	15.67706	21.349	22.00307	30.42412
**5-FU**	57.69034	58.92693	60.08176	65.0792

Table S2 : % of cell growth inhibition of tested compounds with different concentrations in case of hep-g2 cell line.

Code	3.125 µM	6.25 µM	12.5 µM	25 µM
**9a**	30.89938	31.96794	35.88602	36.4203
**9b**	11.84328	23.50846	23.86465	34.81745
**11c**	0	31.43366	31.78985	33.48175
**11d**	30.89938	38.37934	39.26981	39.53695
**14**	35.79697	39.44791	40.16028	43.45503
**16a**	35.35174	37.13268	37.66696	42.38646
**16b**	28.40606	36.33126	40.96171	41.49599
**5-FU**	22.66667	34.4	37.42222	39.37778

Table S3: IC_{50} of the compounds against the two cancer types according to the MTT assay

Compound Code	Structure	IC50 (μM) ± SD	
		**MDA-MB-231**	**HepG2**
**9a**	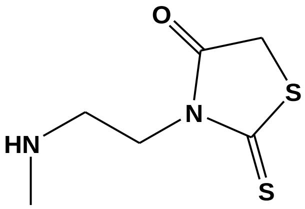	37.7 ± 3.9	27.7± 3.8
**9b**	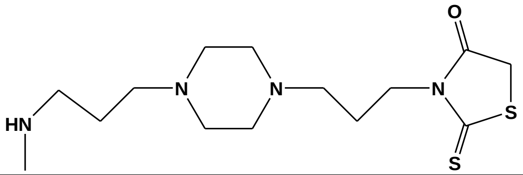	30.0 ± 3.8	36.1±4.2
**11c**	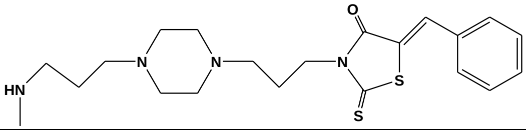	22.7 ± 3.1	35.6±4.1
**11d**	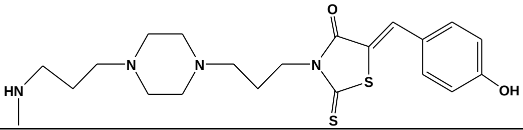	25.3±3.1	25.4± 3.3
**14**	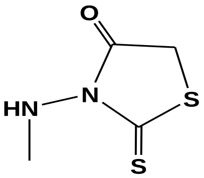	29.9 ± 3.6	23.6±3.8
**16a**	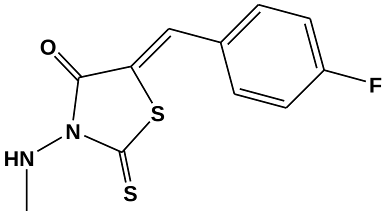	38.8 ± 4.3	24.6± 3.3
**16b**	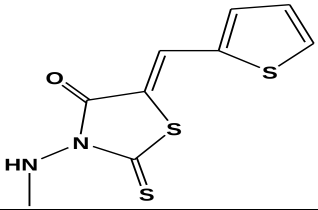	31.9 ± 3.8	25.6±3.1
**5-Fluorouracil**		12.0 ± 2.5	28.0±2.3

Results of flow cytometry analysis

Results of cell cycle analysis in case of HEPG2 cell line:

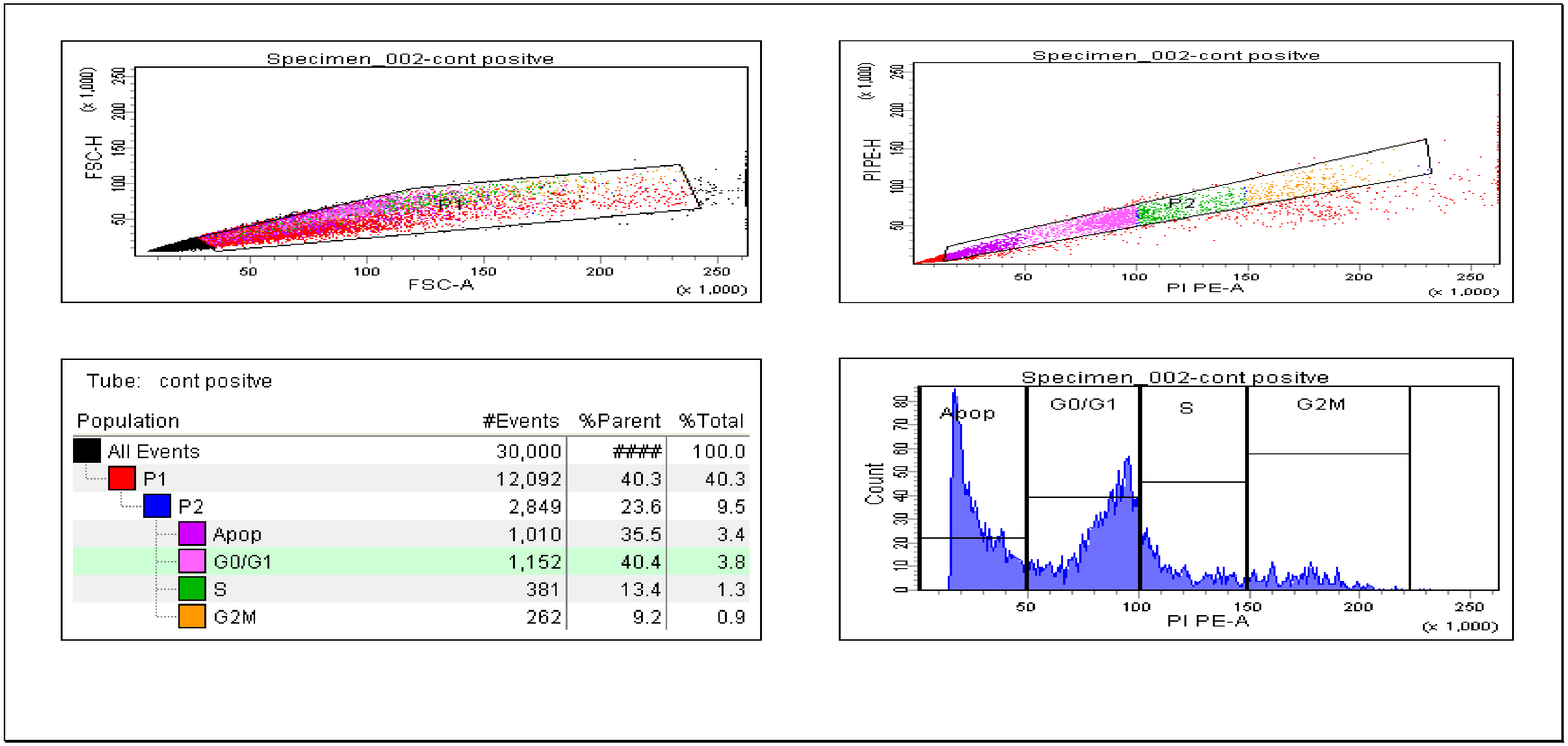

Population	#Events	%Parent	%Total
All Events	30,000	####	100.0
P1	12,092	40.3	40.3
P2	2,849	23.6	9.5
Apop	1,010	35.5	3.4
G0/G1	1,152	40.4	3.8
S	381	13.4	1.3
G2M	262	9.2	0.9

Figure S4: Positive control

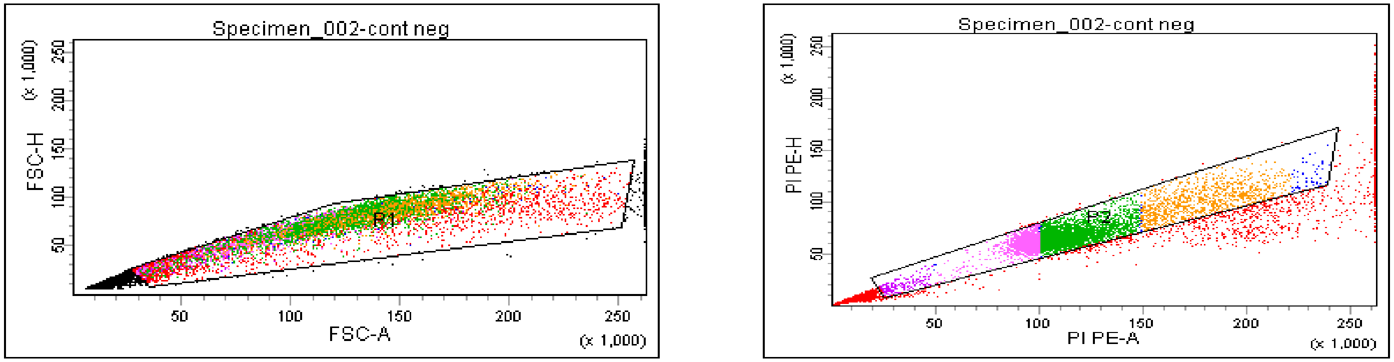

Tube: cont neg

Population	#Events	%Parent	%Total
All Events	16,771	####	100.0
P1	8,460	50.4	50.4
P2	6,046	71.5	36.1
Apop	190	3.1	1.1
G0/G1	1,326	21.9	7.9
S	3,301	54.6	19.7
G2M	1,019	16.9	6.1

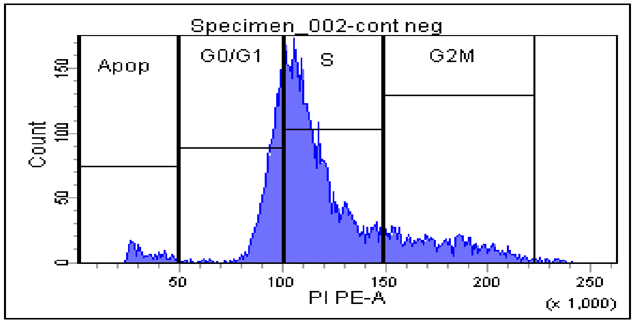

Figure S5: Negative control

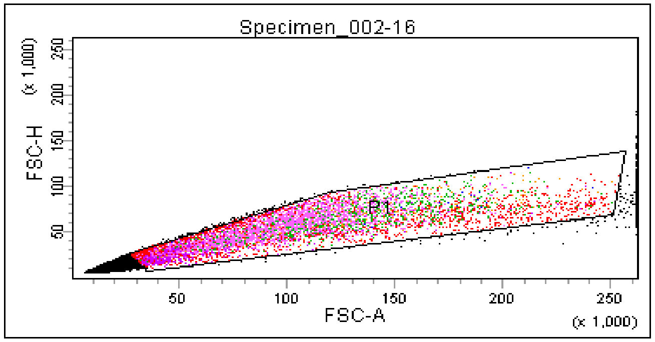

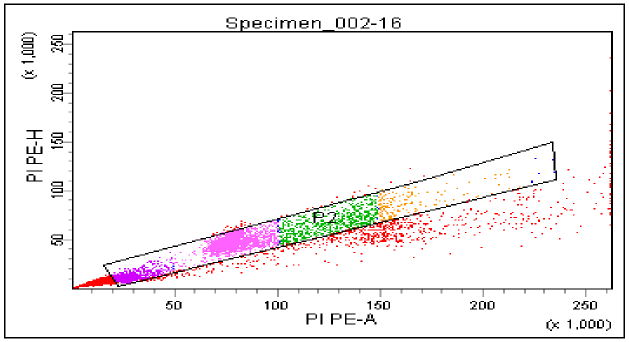

Tube: 16

Population	#Events	%Parent	%Total
All Events	20,000	####	100.0
P1	6,818	34.1	34.1
P2	3,705	54.3	18.5
Apop	620	16.7	3.1
G0/G1	2,256	60.9	11.3
S	638	17.2	3.2
G2M	160	4.3	0.8

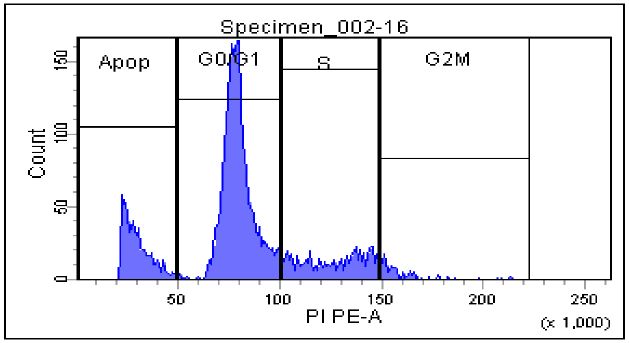

Figure S6: Compound 9a

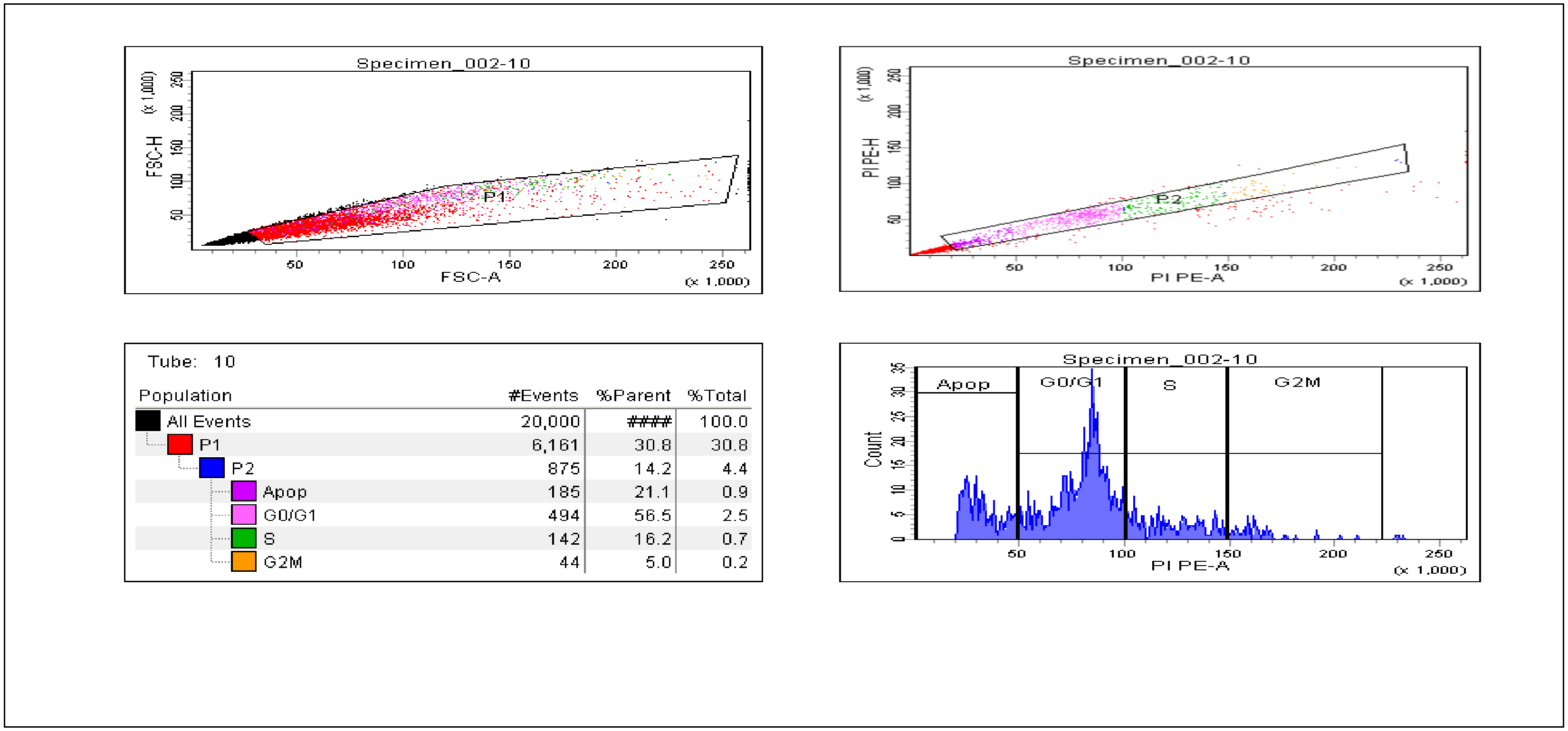

Tube: 10

Population	#Events	%Parent	%Total
All Events	20,000	####	100.0
P1	6,161	30.8	30.8
P2	875	14.2	4.4
Apop	185	21.1	0.9
G0/G1	494	56.5	2.5
S	142	16.2	0.7
G2M	44	5.0	0.2

Figure S7: Compound 11c

Results of cell cycle analysis In case of MDA cell line:

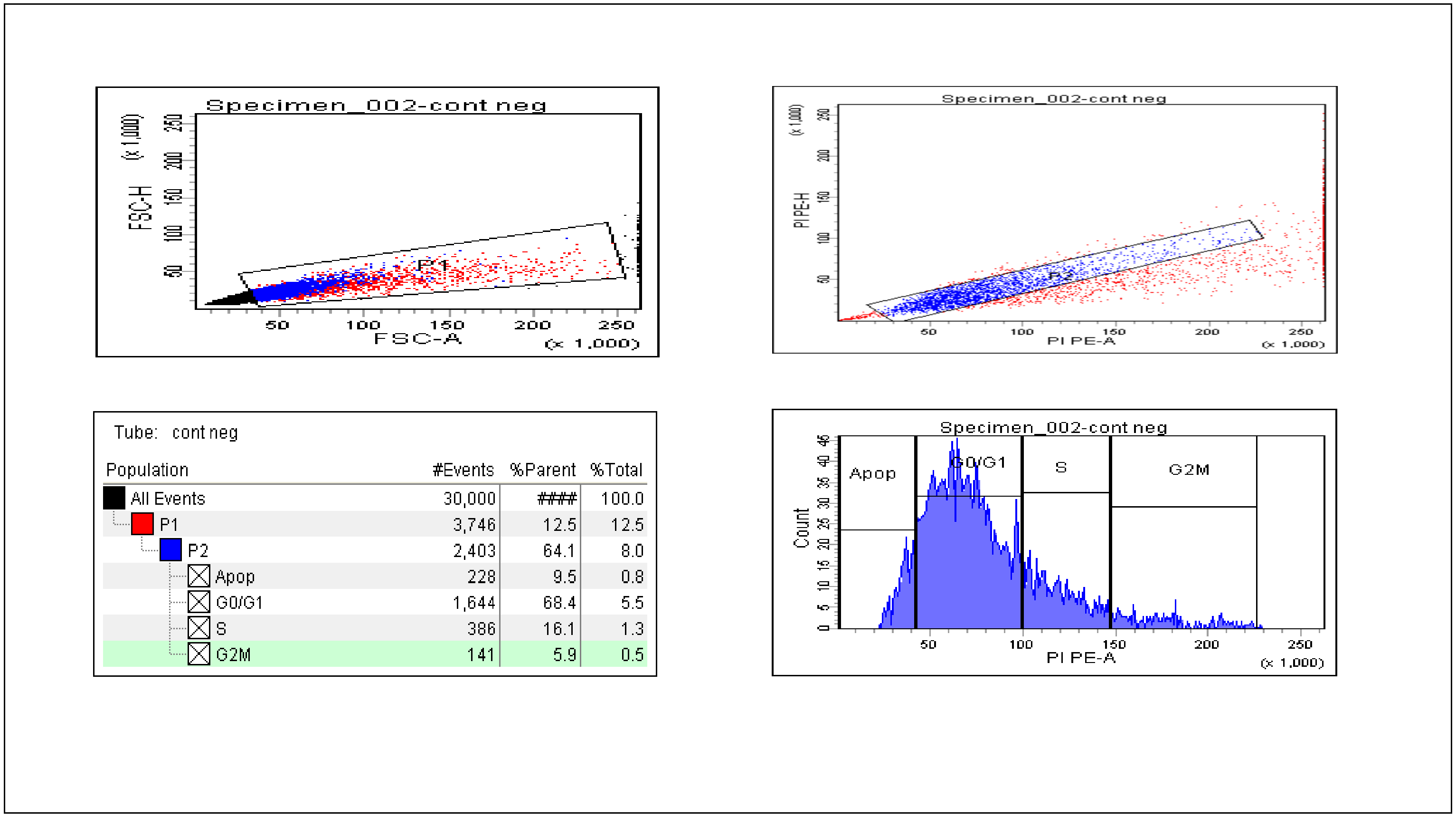

Tube: cont neg

Population	#Events	%Parent	%Total
All Events	30,000	####	100.0
P1	3,746	12.5	12.5
P2	2,403	64.1	8.0
Apop	228	9.5	0.8
G0/G1	1,644	68.4	5.5
S	386	16.1	1.3
G2M	141	5.9	0.5

Figure S8: Negative Control

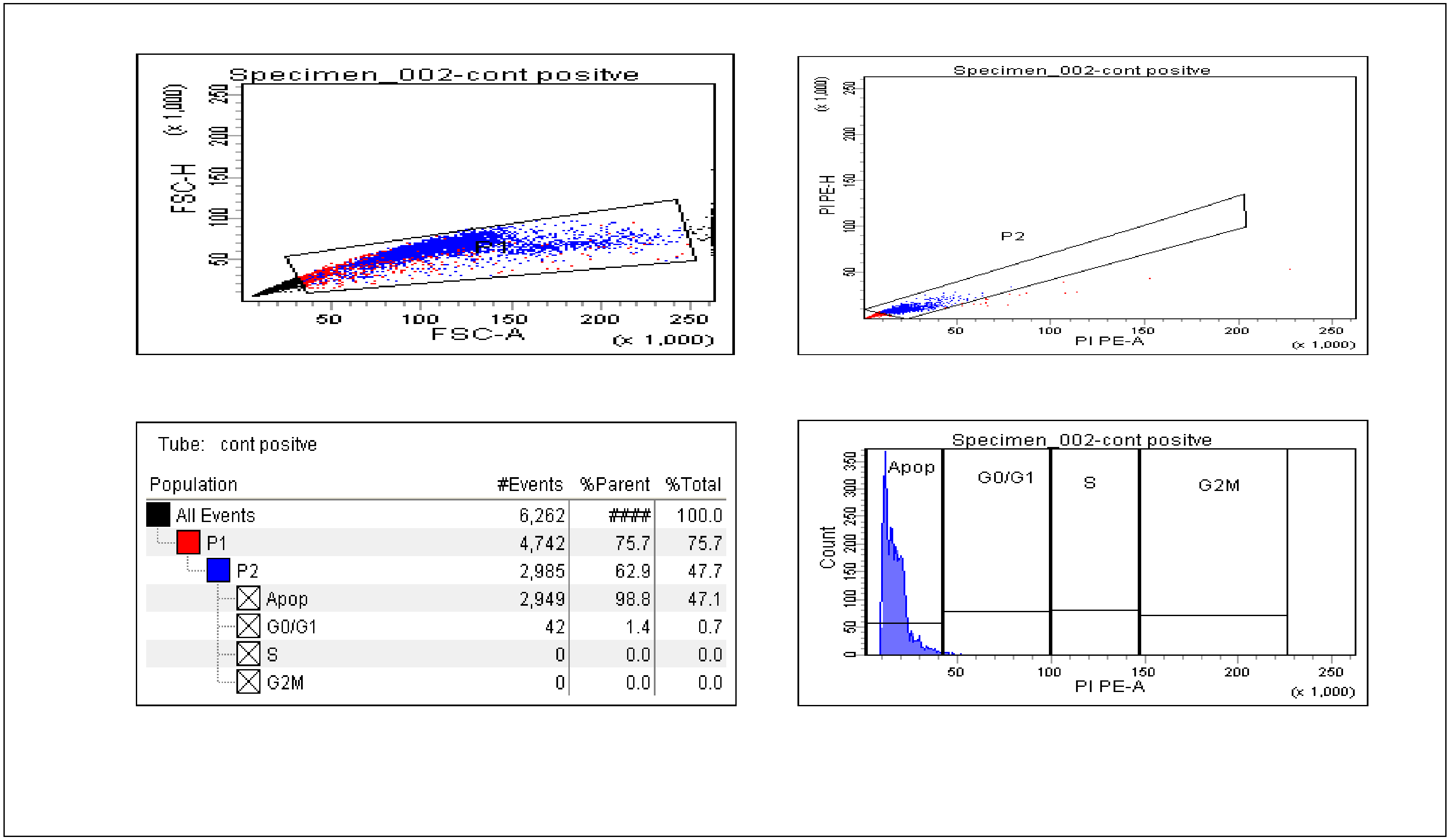

Tube: cont positve

Population	#Events	%Parent	%Total
All Events	6,262	####	100.0
P1	4,742	75.7	75.7
P2	2,985	62.9	47.7
Apop	2,949	98.8	47.1
G0/G1	42	1.4	0.7
S	0	0.0	0.0
G2M	0	0.0	0.0

Figure S9: Positive control

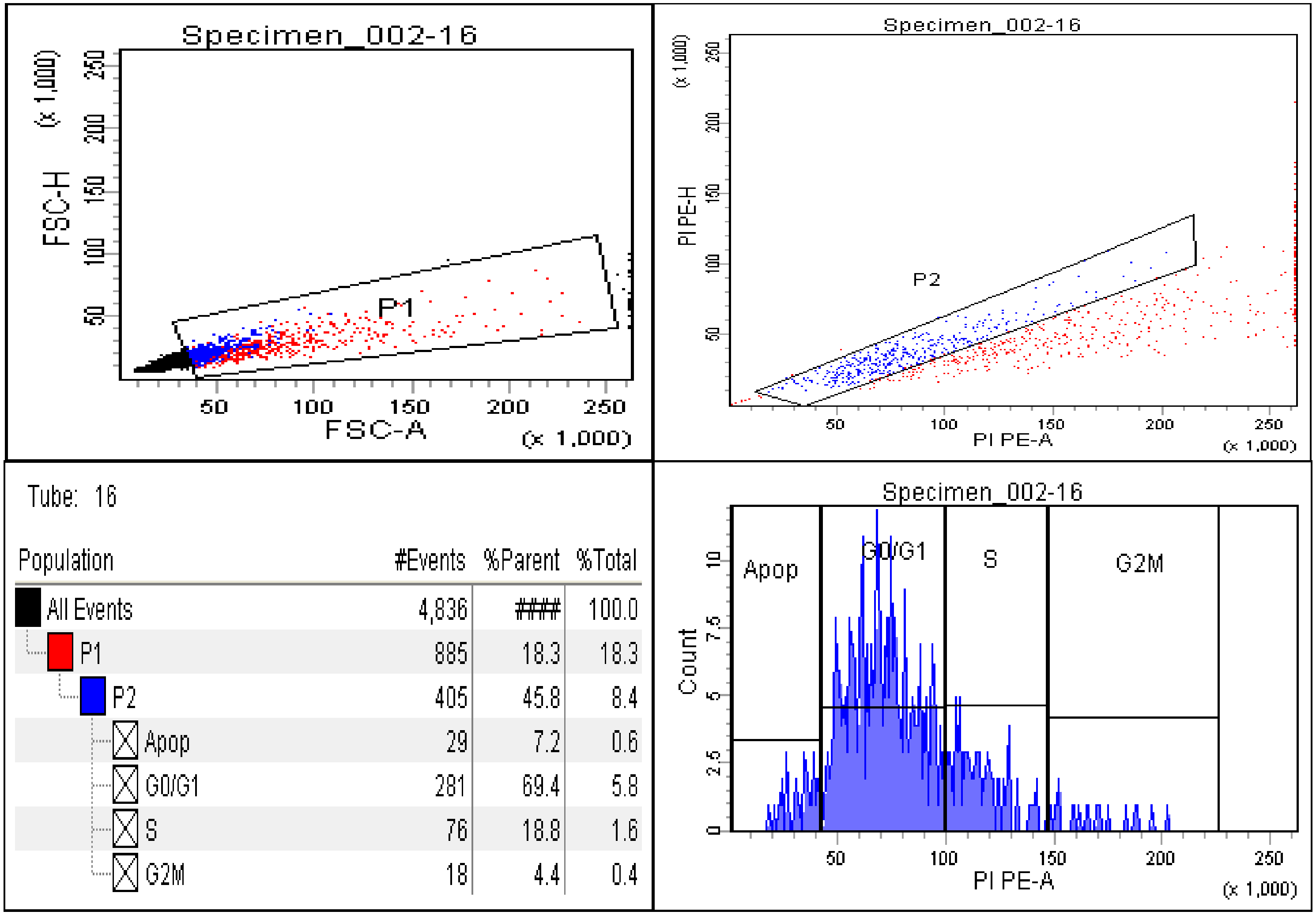

Population	#Events	%Parent	%Total
All Events	4,836	####	100.0
P1	885	18.3	18.3
P2	405	45.8	8.4
Apop	29	7.2	0.6
G0/G1	281	69.4	5.8
S	76	18.8	1.6
G2M	18	4.4	0.4

Figure S10: Compound **9a**

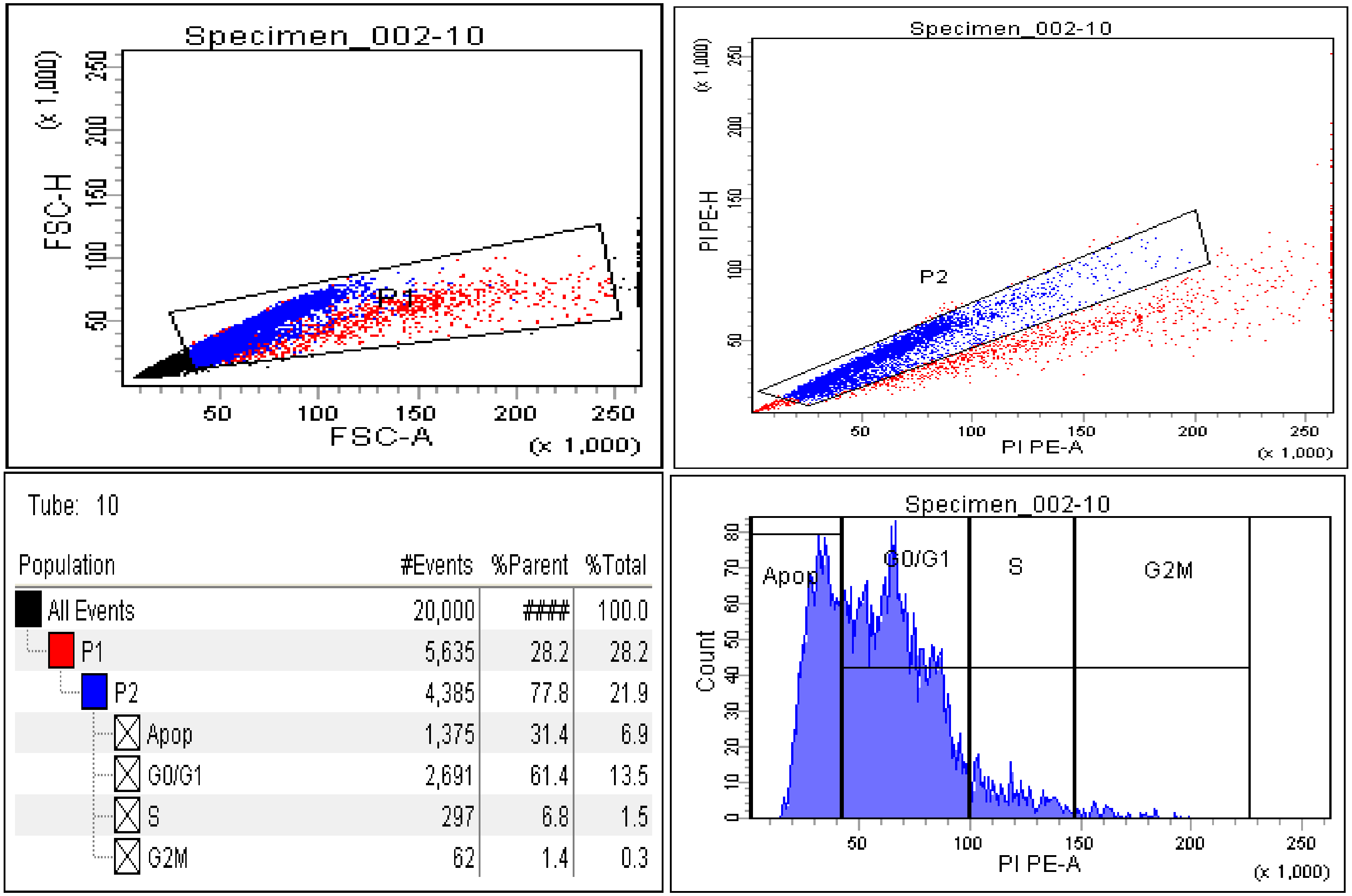

Population	#Events	%Parent	%Total
All Events	20,000	####	100.0
P1	5,635	28.2	28.2
P2	4,385	77.8	21.9
Apop	1,375	31.4	6.9
G0/G1	2,691	61.4	13.5
S	297	6.8	1.5
G2M	62	1.4	0.3

Figure S11: compound**11c**

Apoptosis analysis in case of Hep-G2

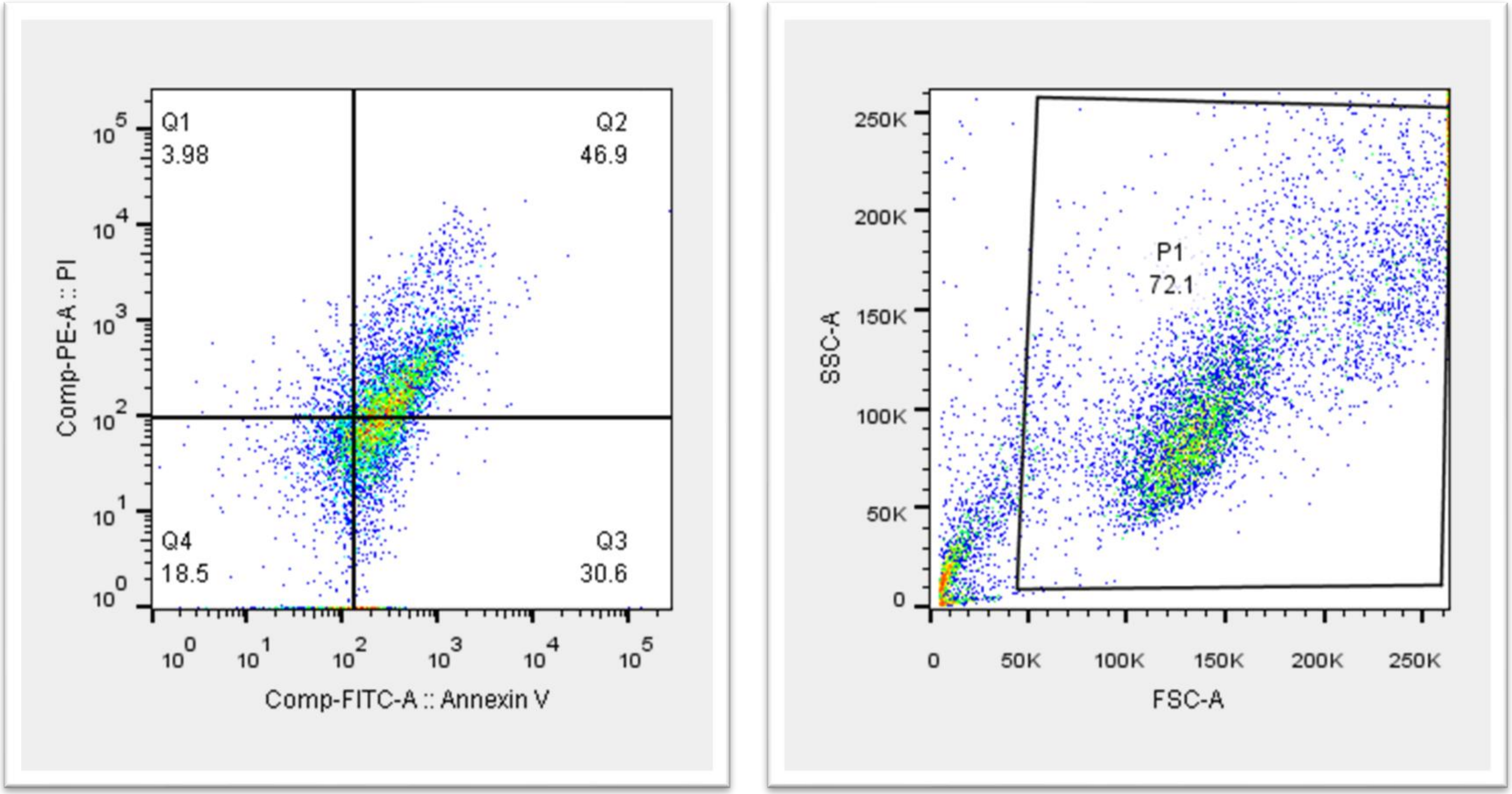

Figure S12: Positive Control

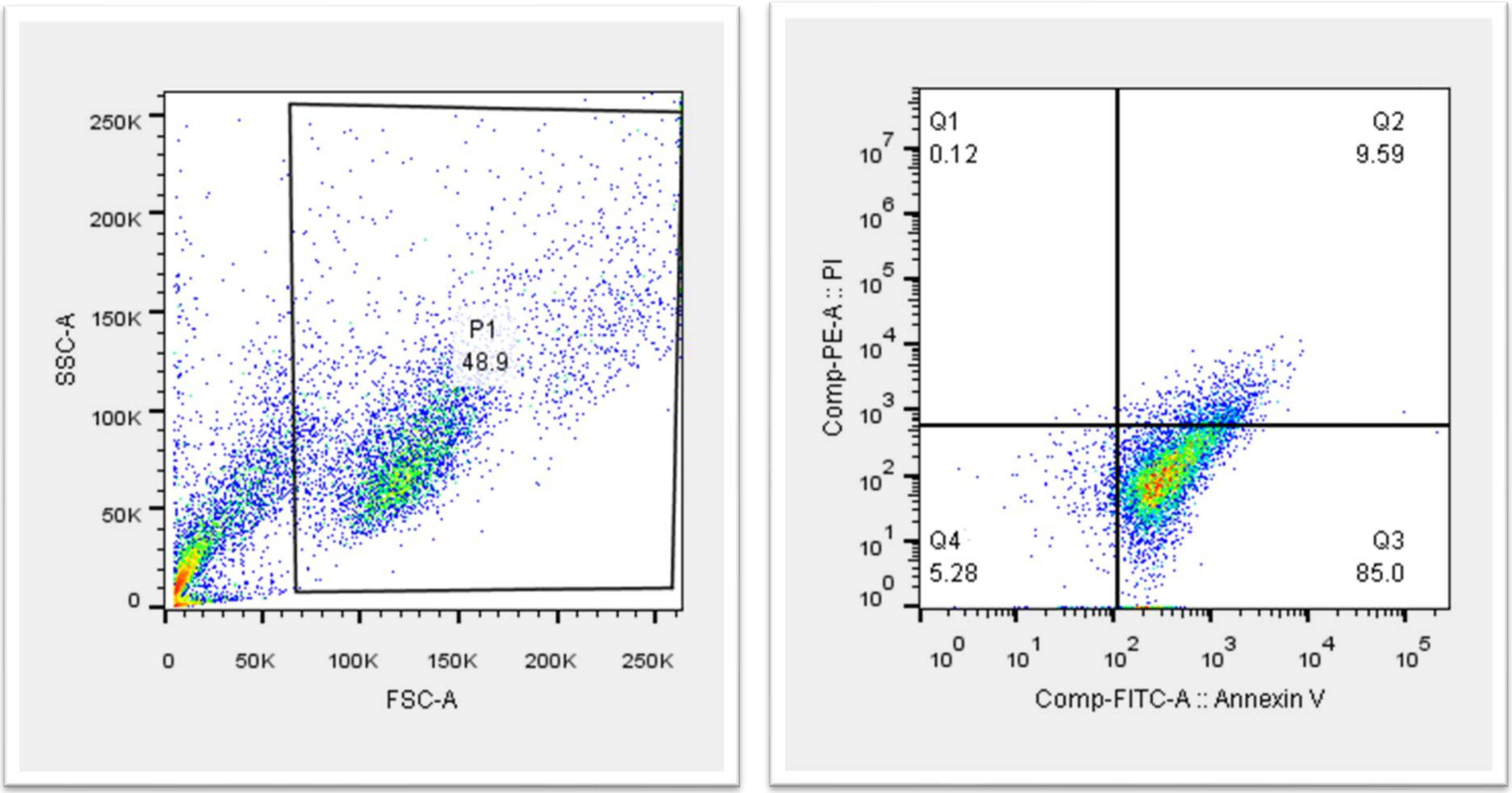

Figure S13: Negative Control

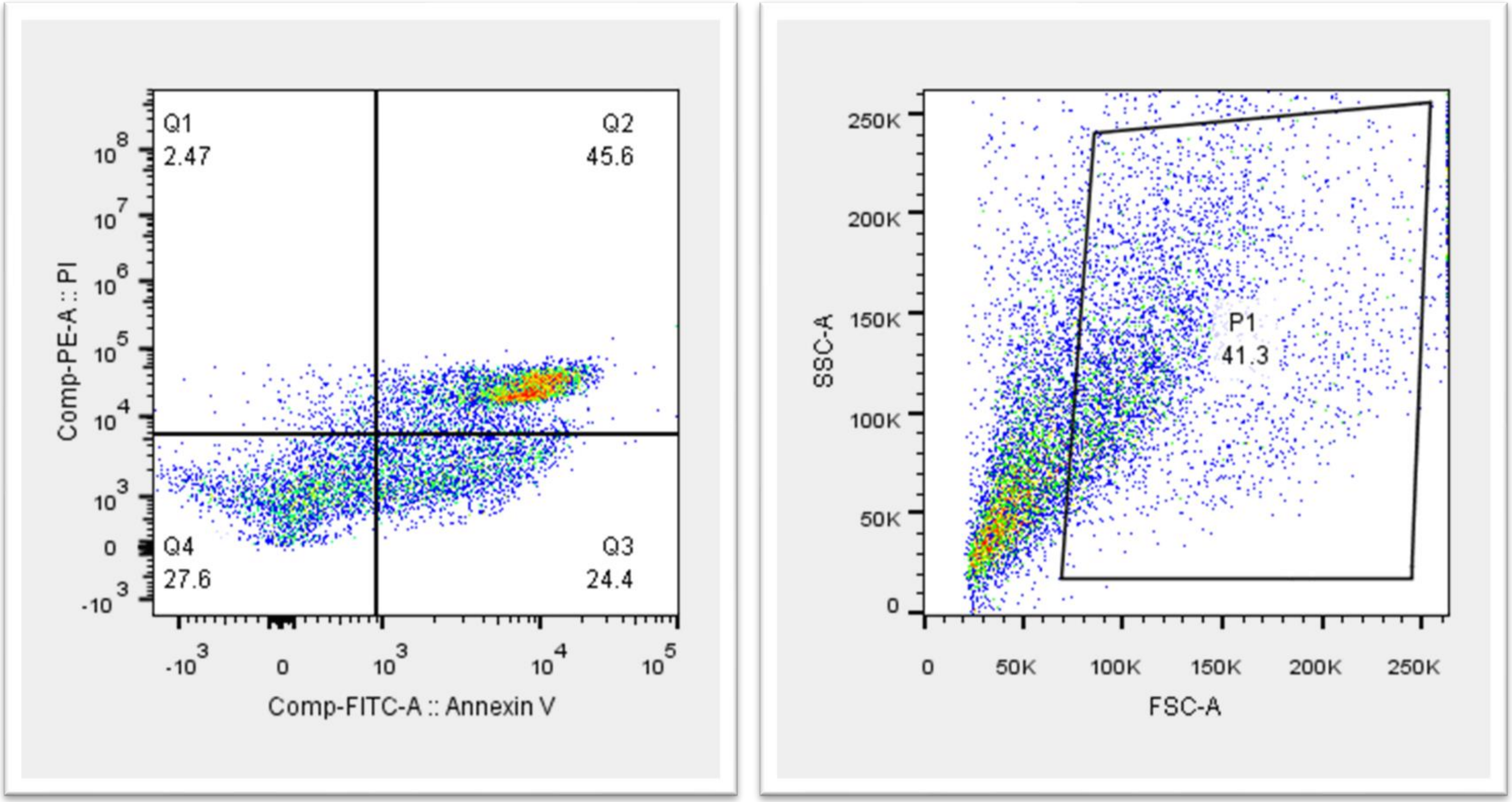

Figure S14: Compound **9a**

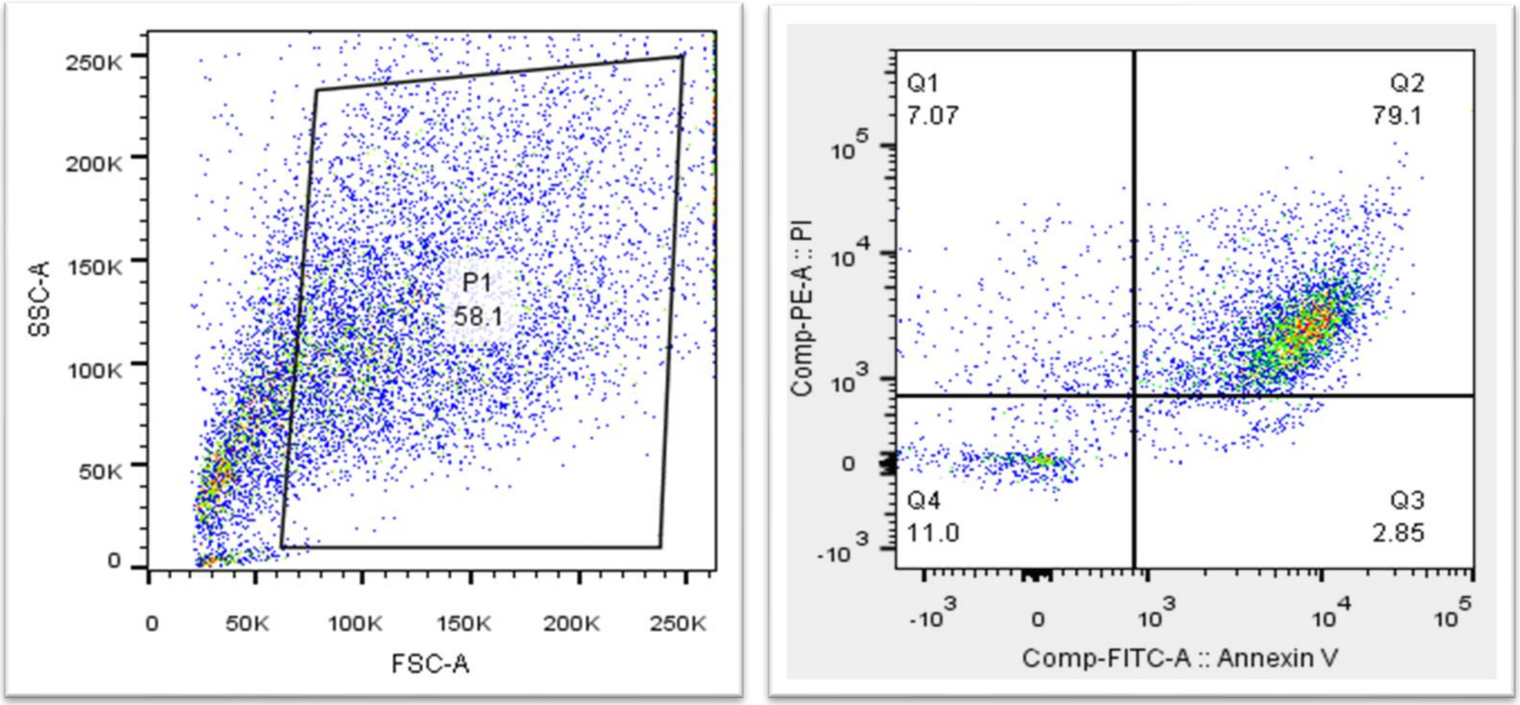

Figure S15: Compound **11c**

Apoptosis analysis in case of MDA cell line:

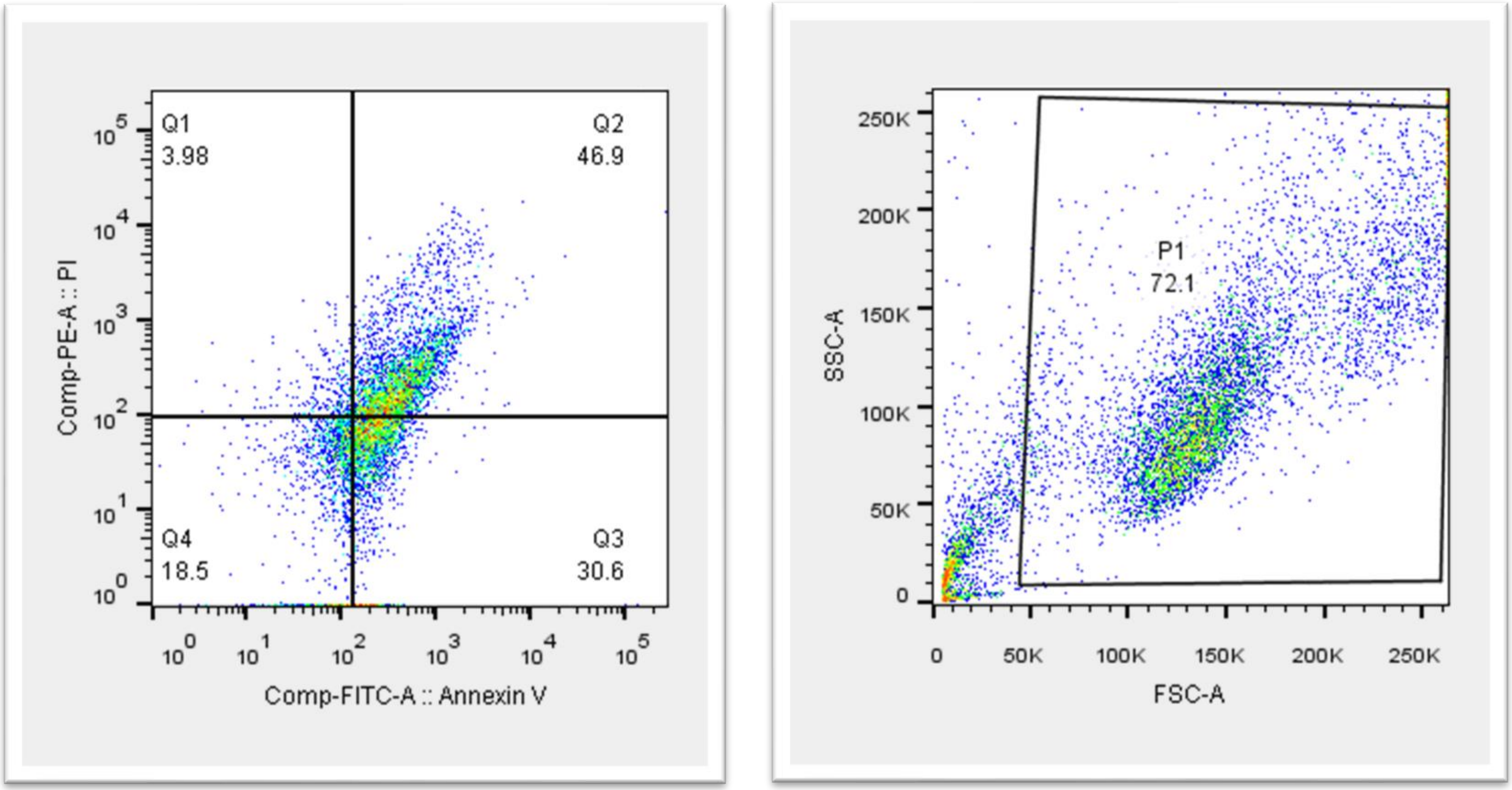

Figure S16: Positive Control

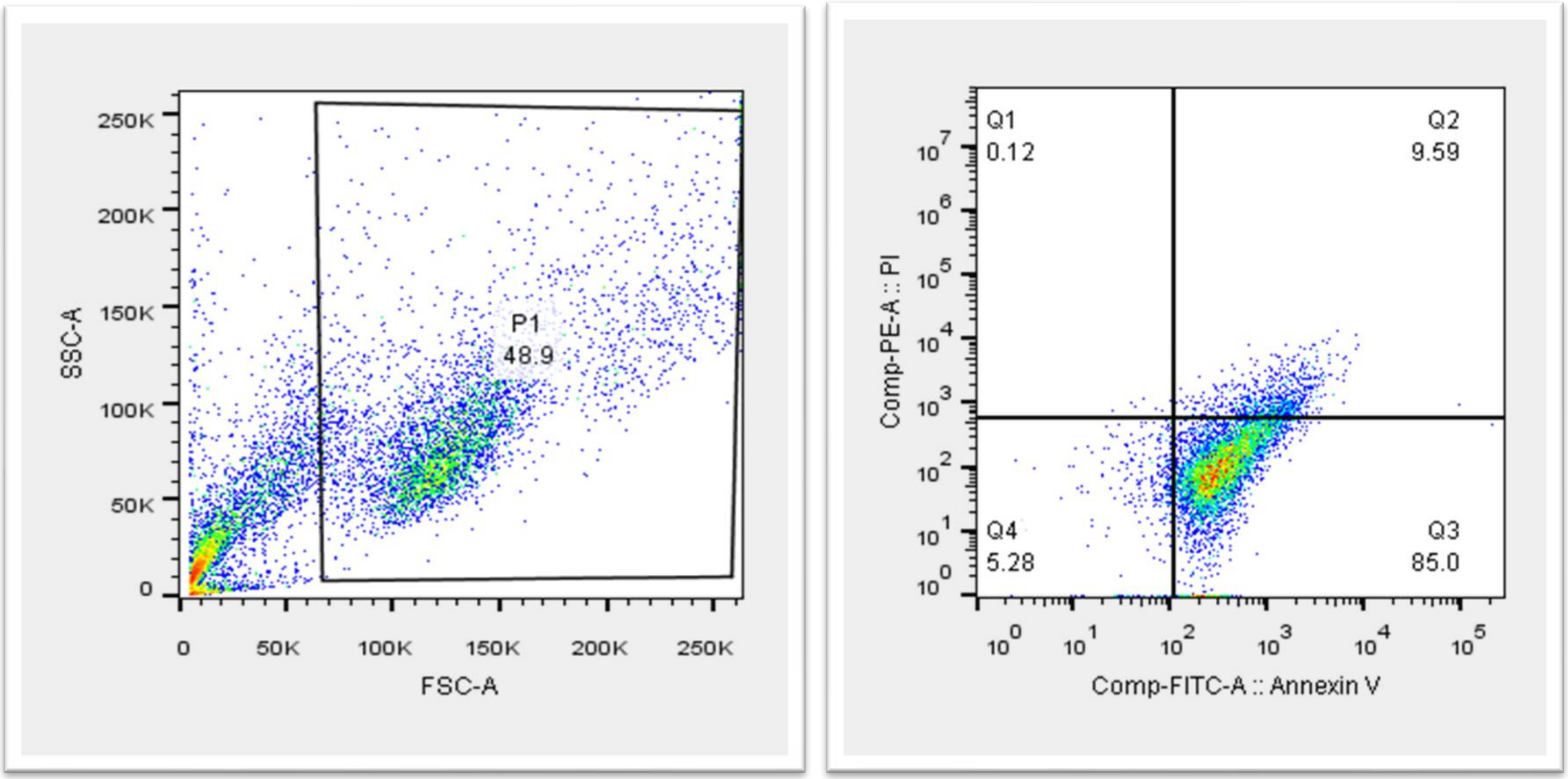

Figure S17: Negative Control

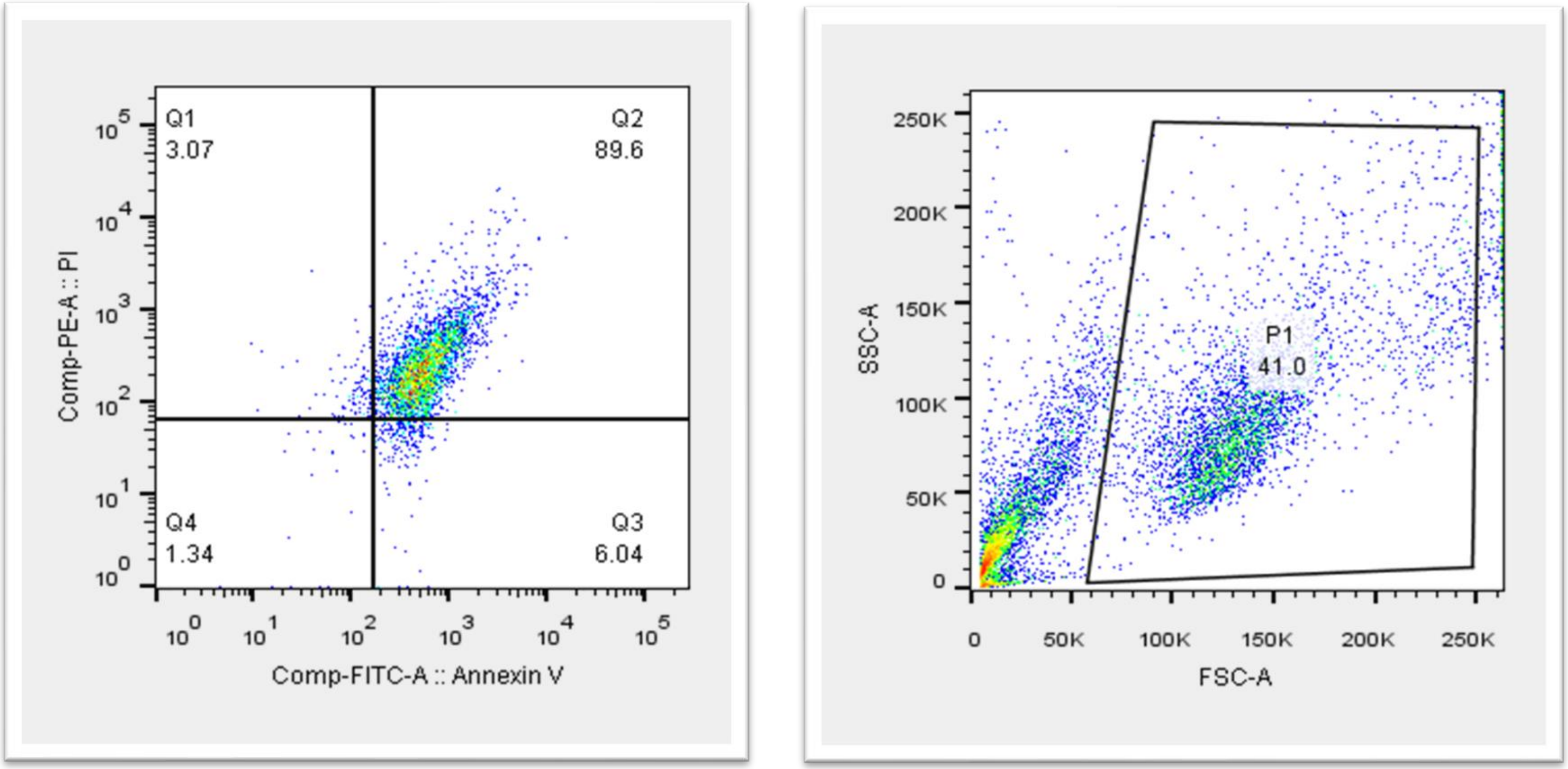

Figure S18: Compound **9a**

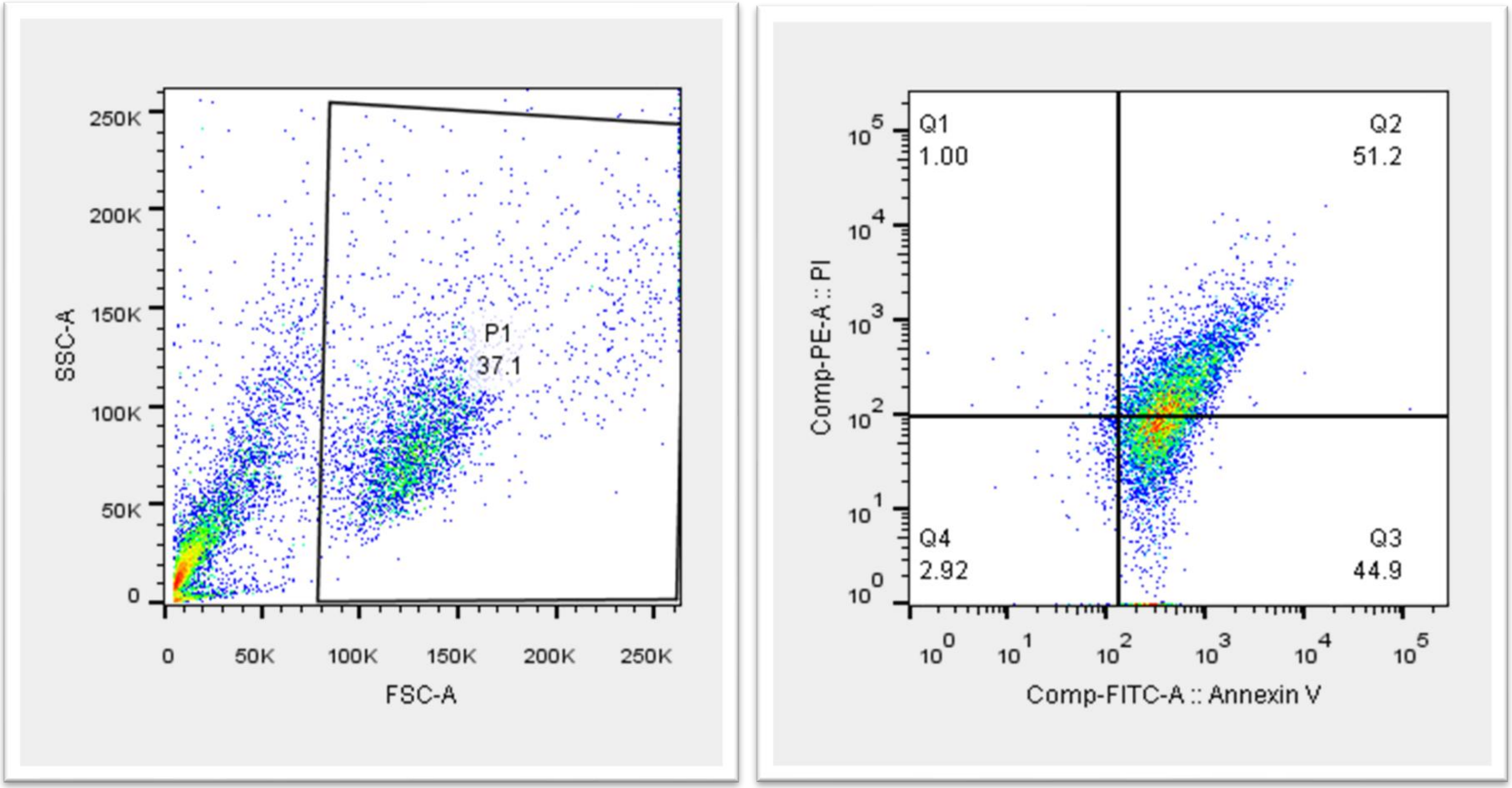

Figure S19: Compound **11c**

Table S4: concentrations in case of hepg2

Compound	IC_{50}	v of compd + complete media µ
**9a**	0.7	2 +2998µ
**11c**	0.7	2 +2998µ
**5FU**	0.1	0.78+2999µ

Table S5: concentrations in case of MDA

Compound code	IC_{50}	v of compd + complete media µ
**9a**	0.41	1.2+2998.8µ
**11c**	0.47	1.4+2998.6µ
**5FU**	0.1	0.78+2.999µ

Table S6: Results of cell cycle and apoptosis analysis.

compound	Cell cycle								apoptosis					
	HEPG2				MDA				HEPG2			MDA		
	Apoptosis	G0/G1	S phase	G2/M	Apoptosis	G0/G1	S phase	G2/M	Q1	Q2	Q3	Q1	Q2	Q3
**9a**	16.7	80.9	17.2	4.3	7.2	69.4	18.8	4.4	2.47	45.6	24.4	3.07	89.6	6.04
**11c**	21.1	56.5	16.2	5	31.4	61.4	6.8	1.4	7.07	45.6	24.4	1	51.2	44.9
Positive Control	35.5	40.4	13.4	9.2	98.8	1.4	0	0	4.68	83.8	8.74	3.98	46.9	30.6
Negative Control	3.1	21.9	54.6	16.9	9.5	68.4	16.1	5.9	0.22	1.46	42.2	0.12	9.59	85

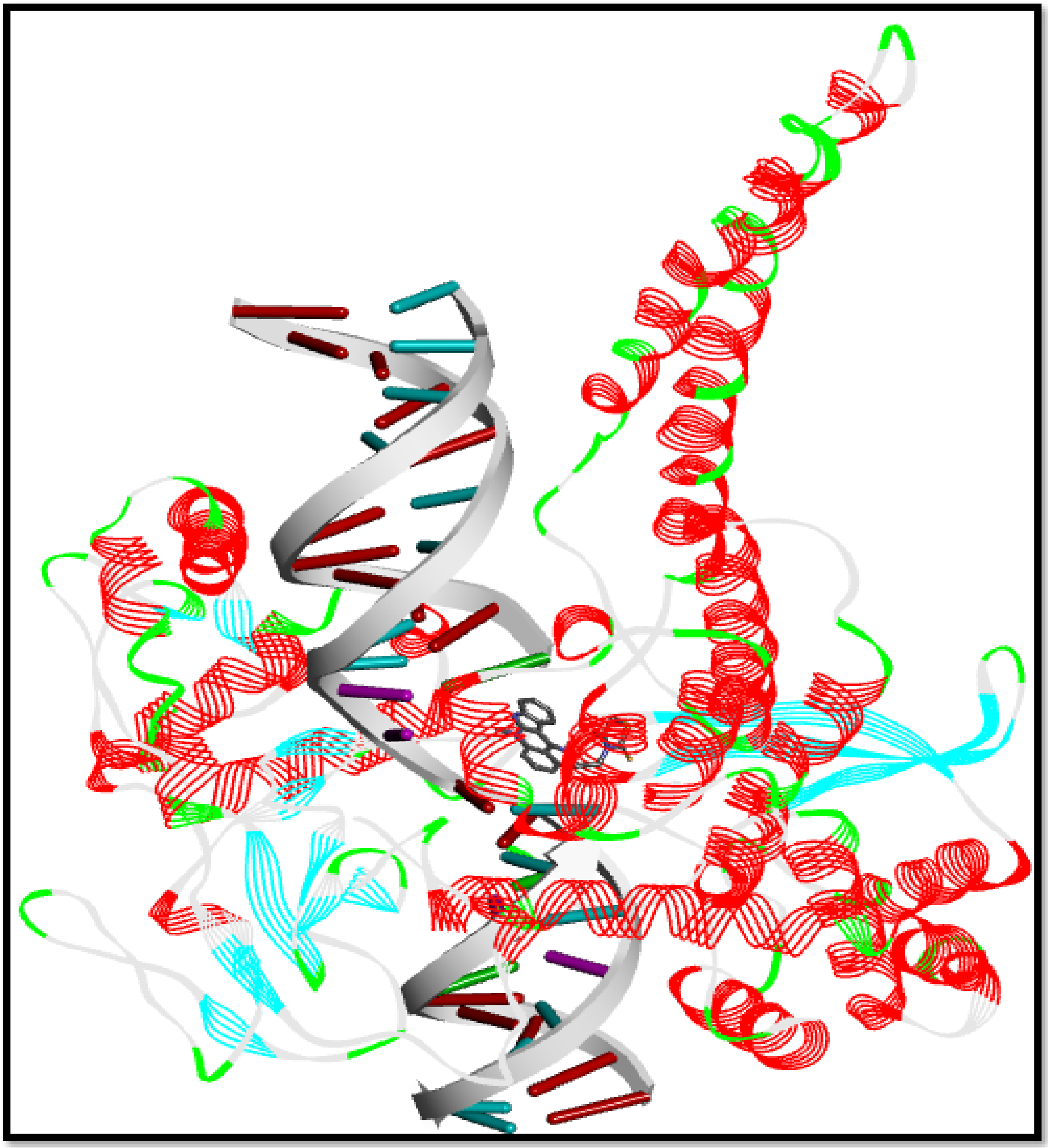

Figure S20a: docked complex of Topoisomerase I enzyme and compound **9a**.

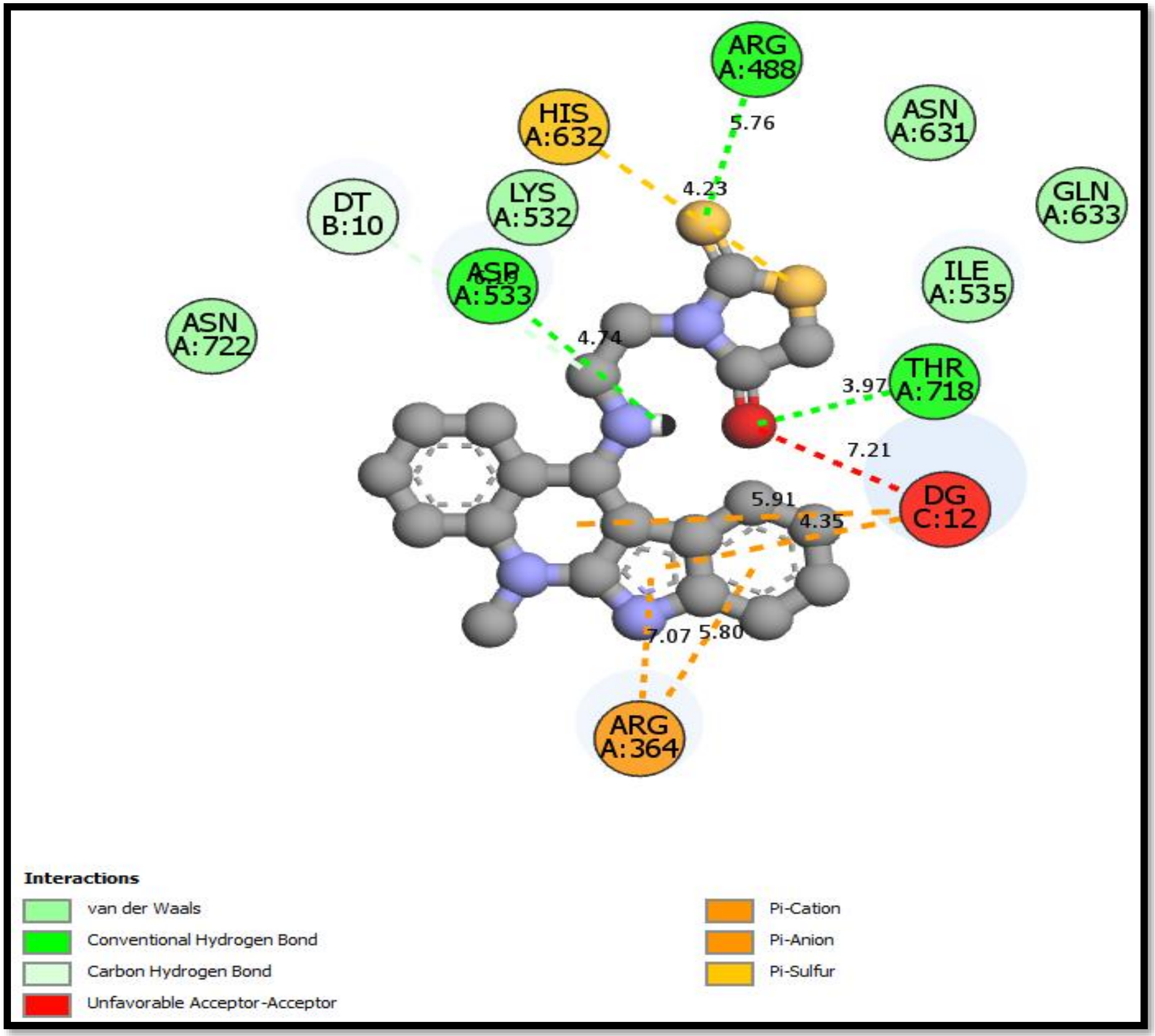

Figure S20b: docked complex of Topoisomerase I enzyme and compound **9a** interactions.

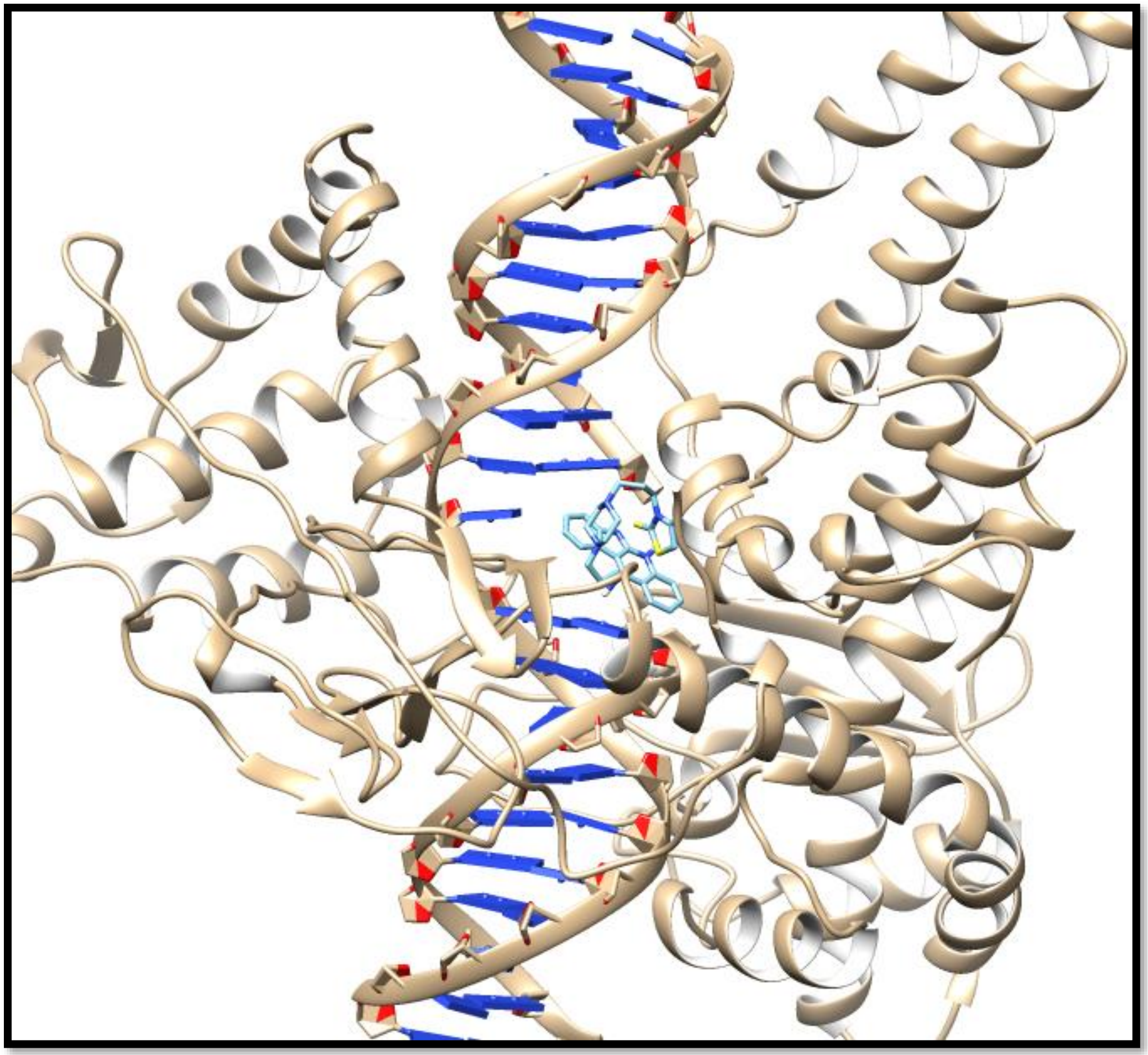

Figure S21a: docked complex of Topoisomerase I enzyme and compound **9b**.

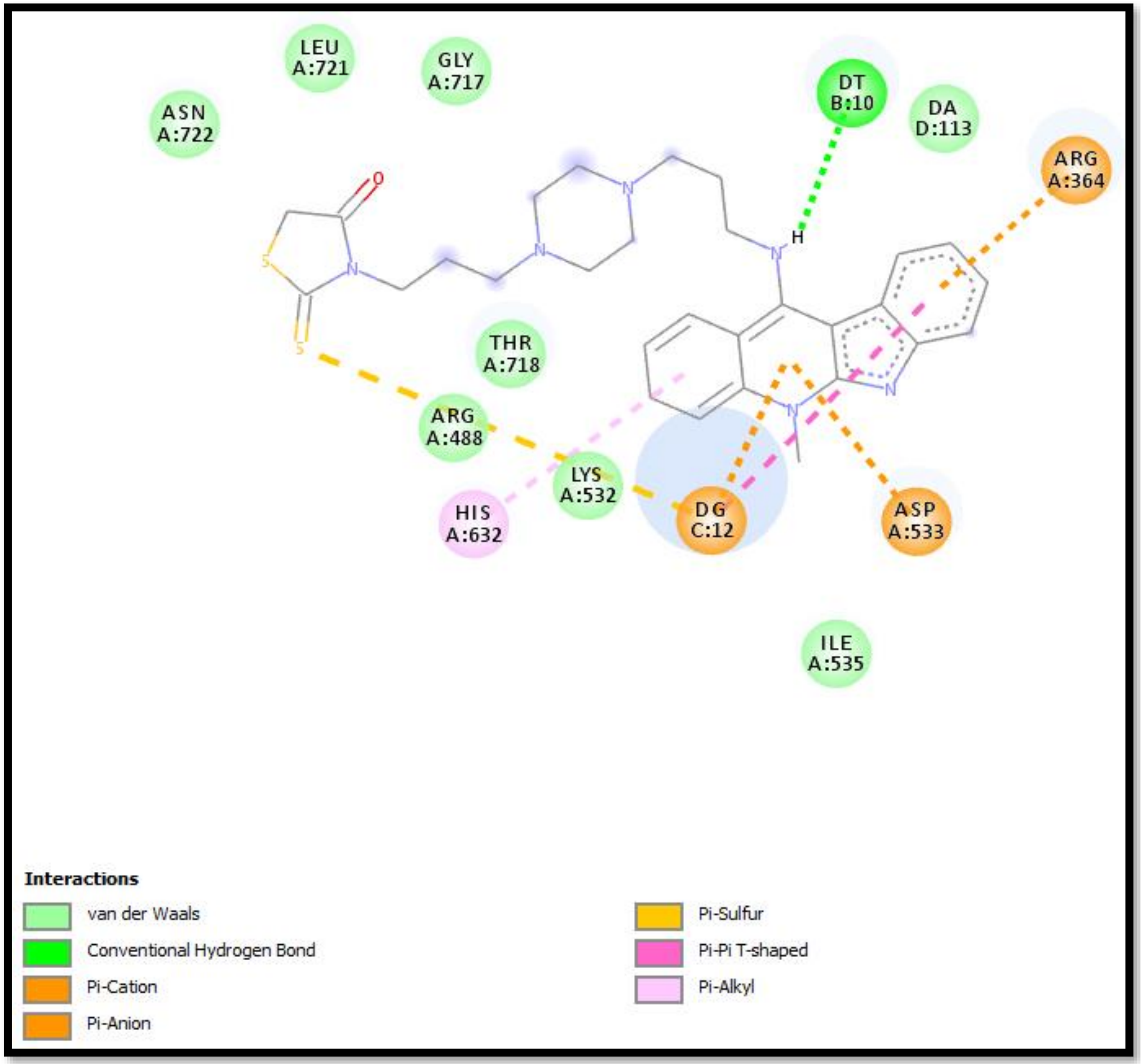

Figure S21b: docked complex of Topoisomerase I enzyme and compound **9b** interactions.

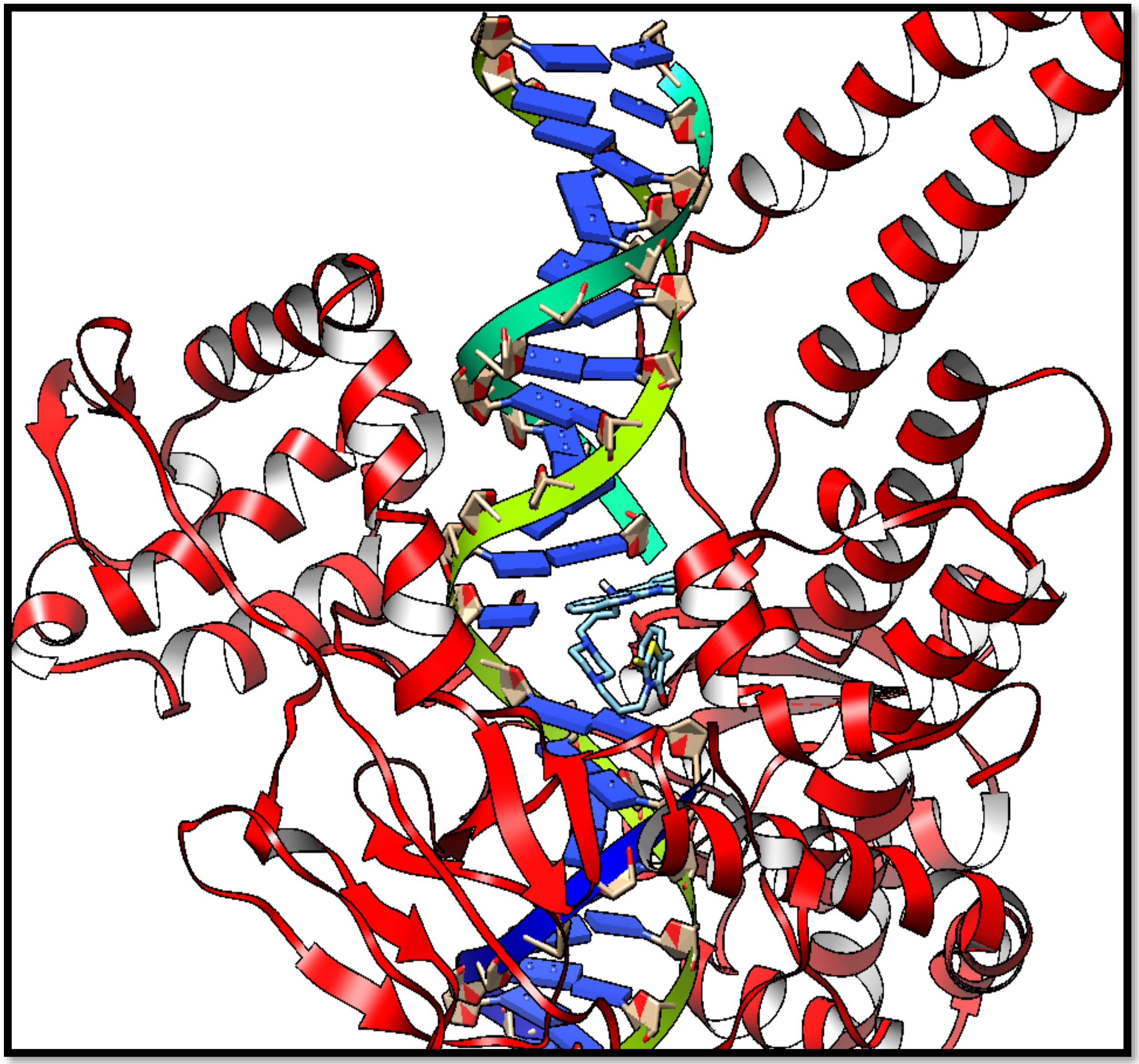

Figure S22a: docked complex of Topoisomerase I enzyme and compound **11c**.

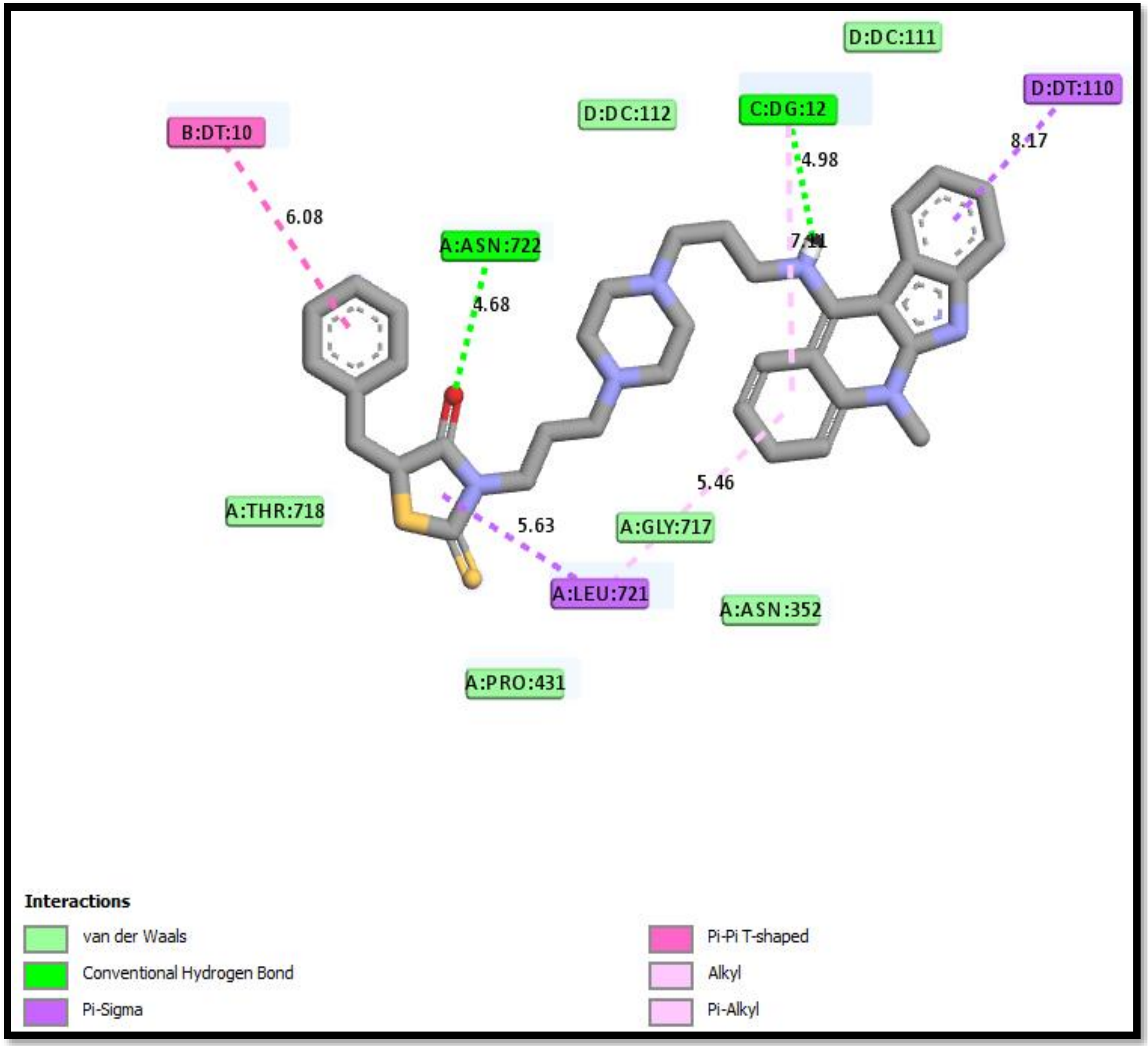

Figure S22b: docked complex of Topoisomerase I enzyme and compound **11c** interactions.

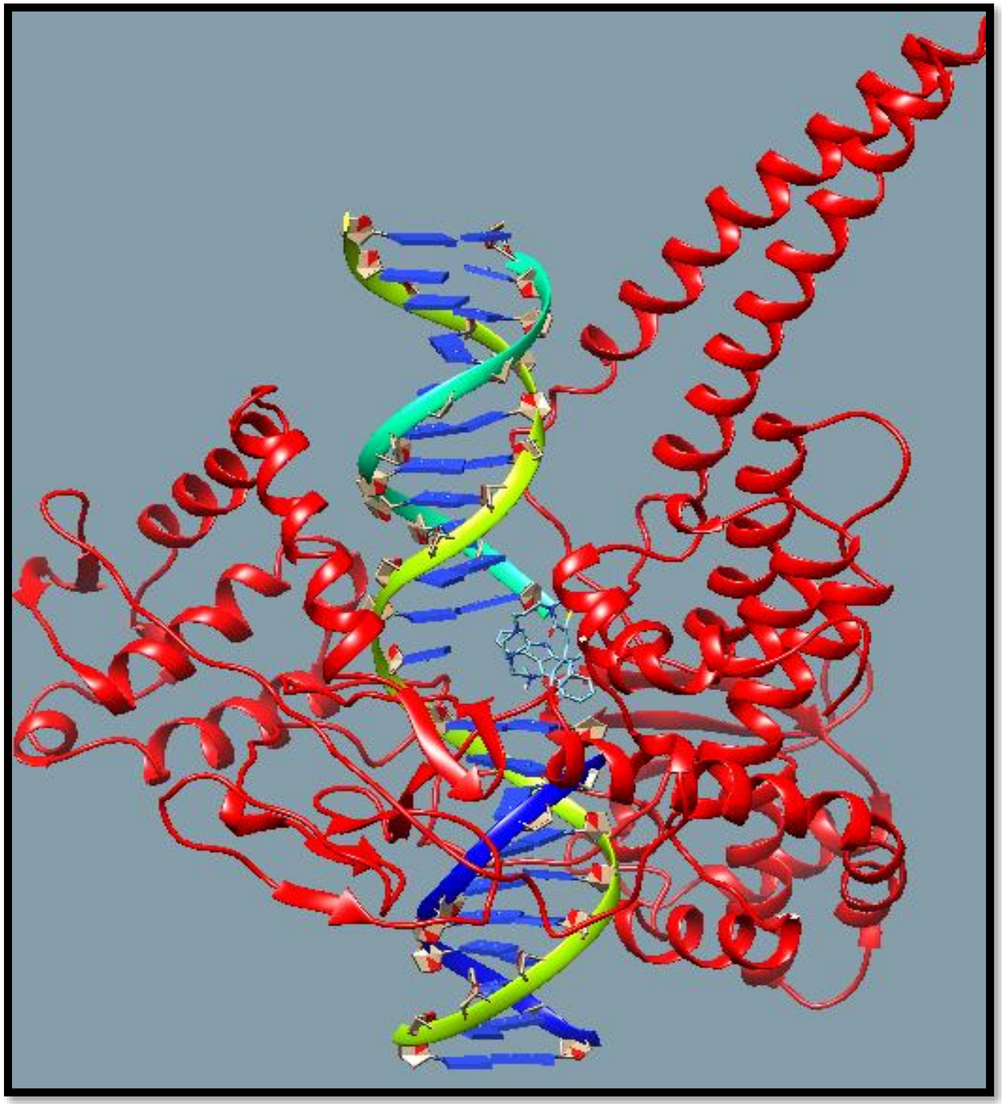

Figure S23a: docked complex of Topoisomerase I enzyme and compound **11d**.

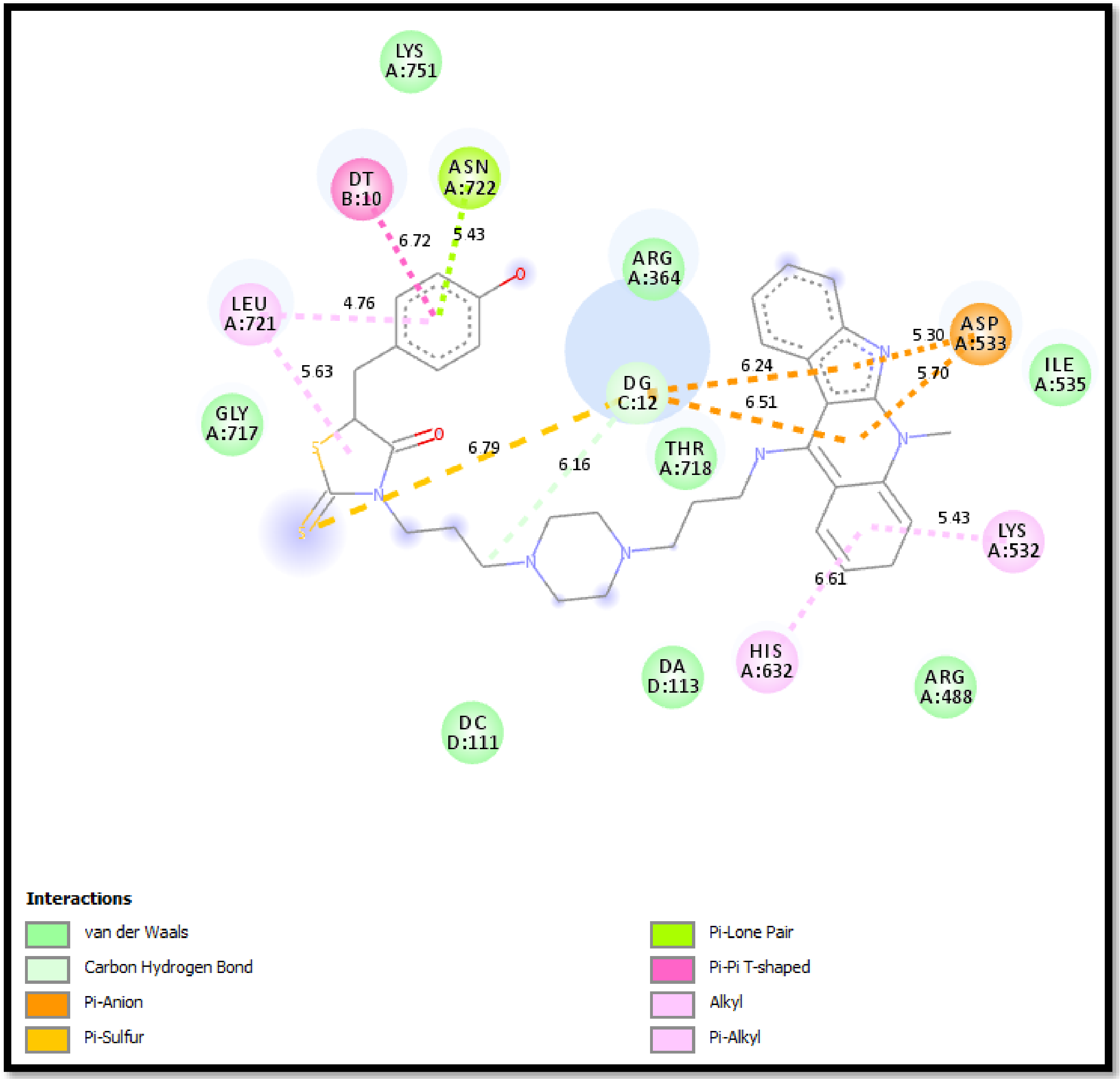

Figure S23b: docked complex of Topoisomerase I enzyme and compound **11d** interactions.

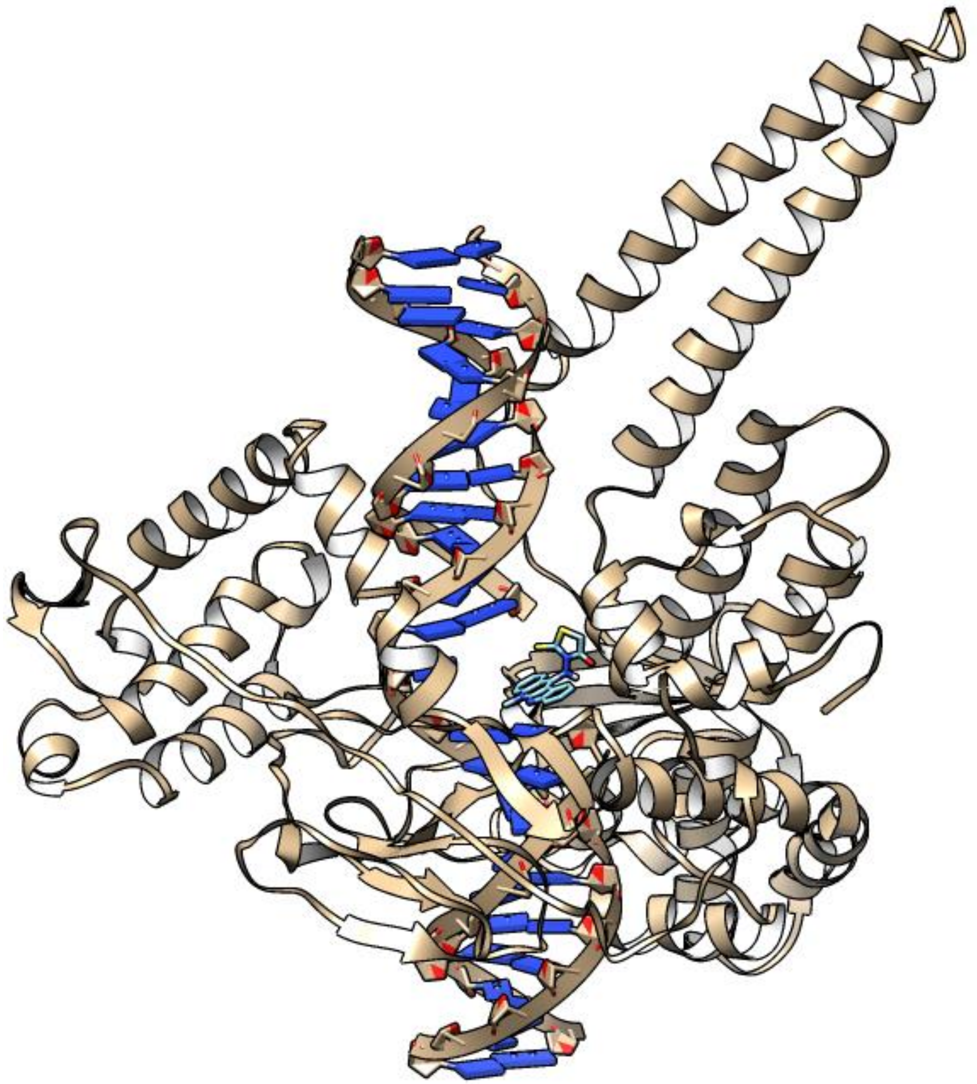

Figure S24a: docked complex of Topoisomerase I enzyme and compound **14**.

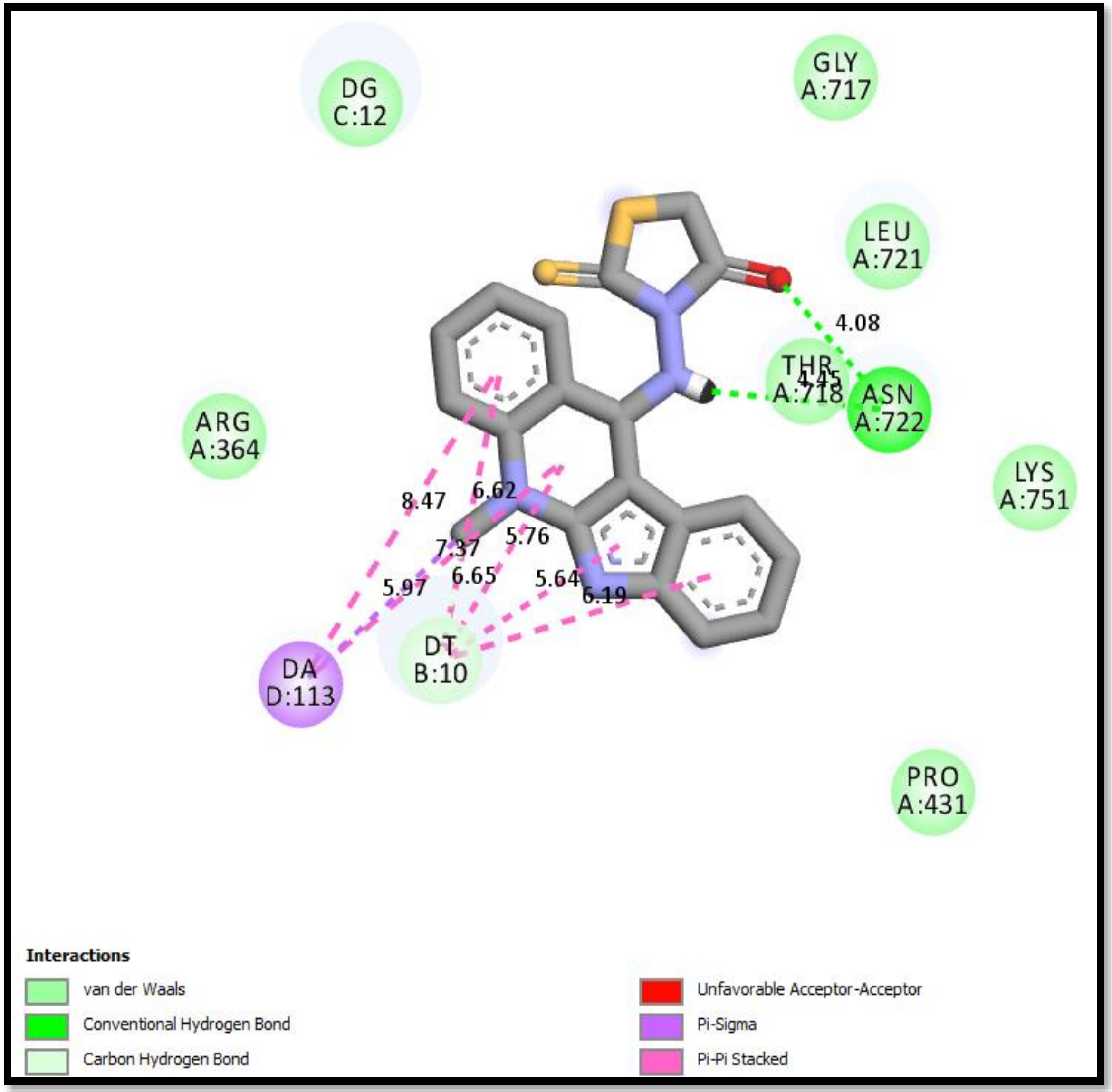

Figure S24b: docked complex of Topoisomerase I enzyme and compound **14** interactions.

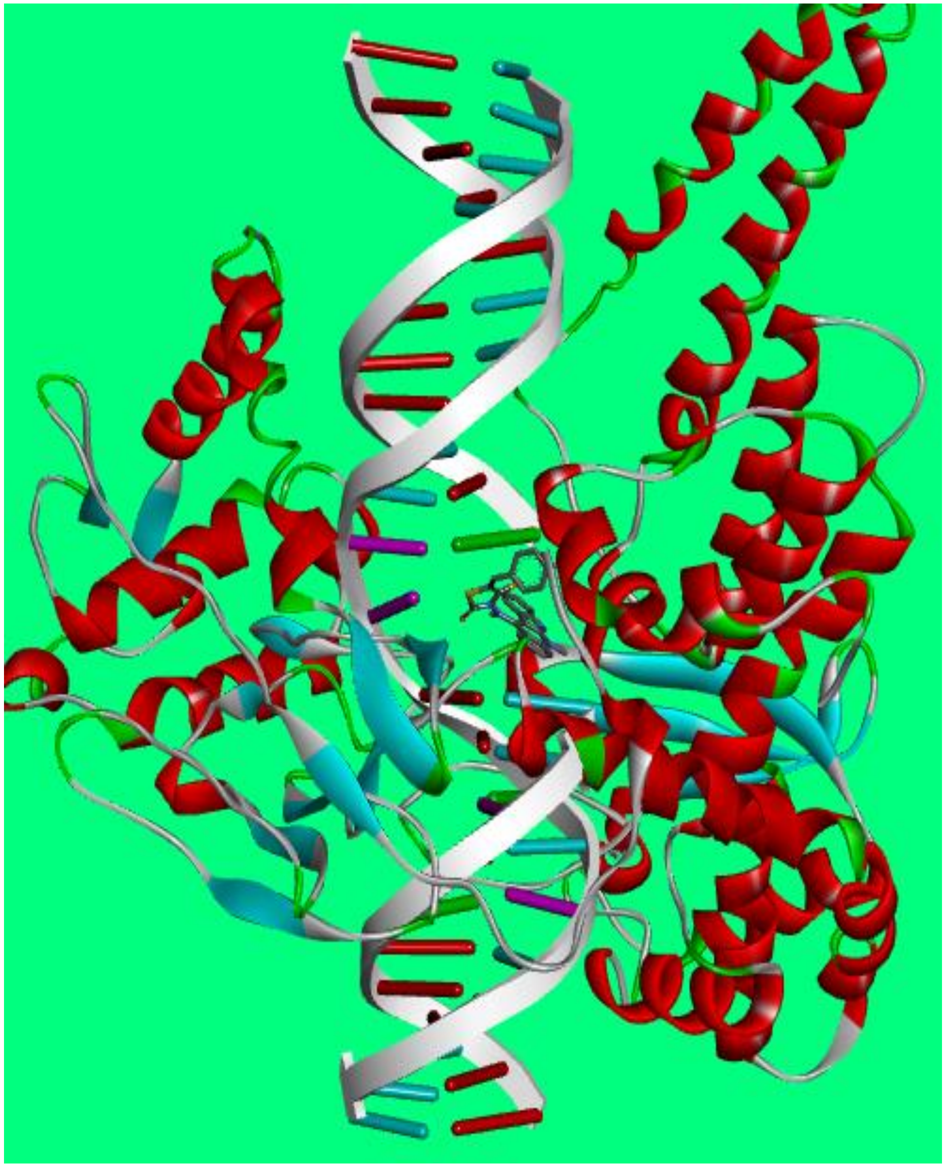

Figure S25a: docked complex of Topoisomerase I enzyme and compound **16a**.

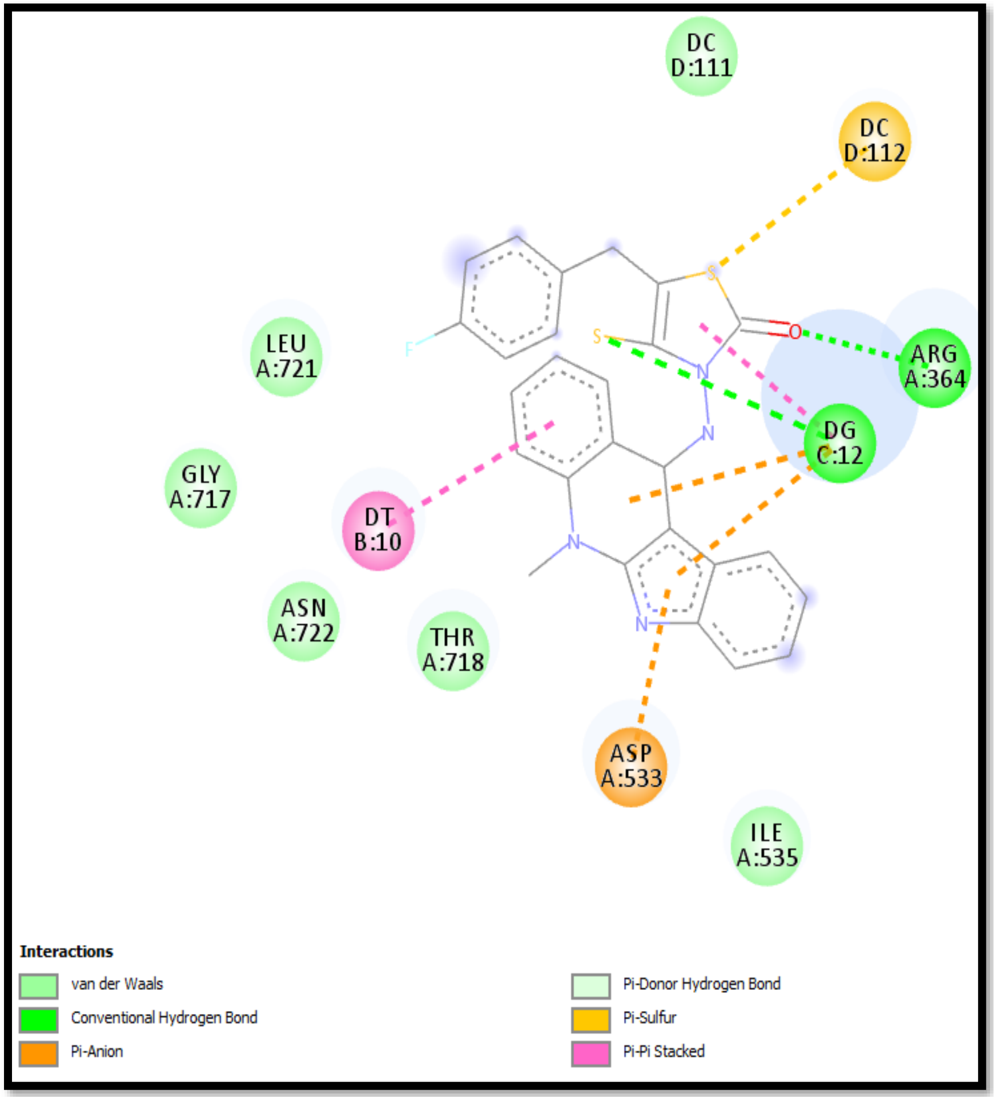

Figure S25b: docked complex of Topoisomerase I enzyme and compound **16a** interactions.

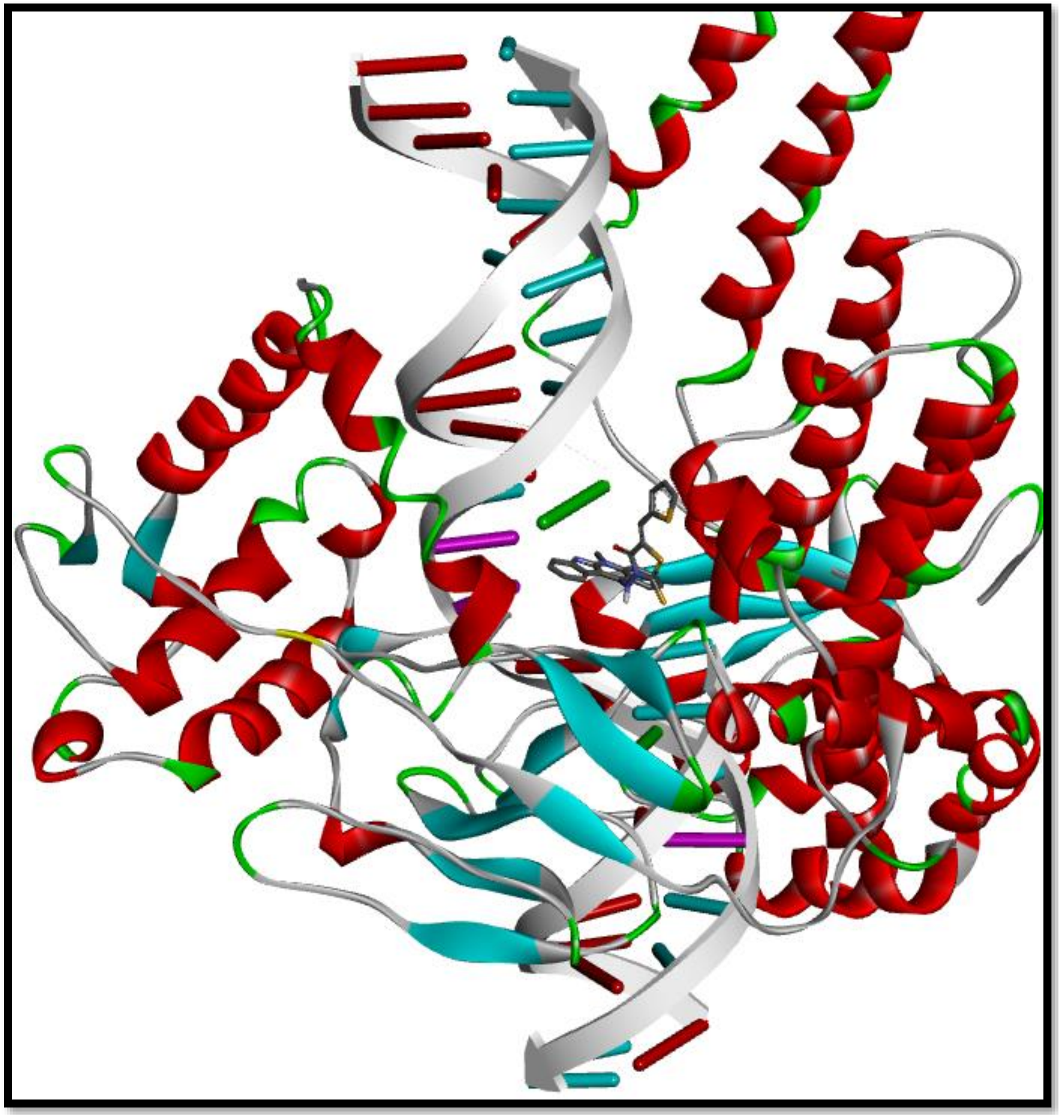

Figure S26a: docked complex of Topoisomerase I enzyme and compound **16b** .

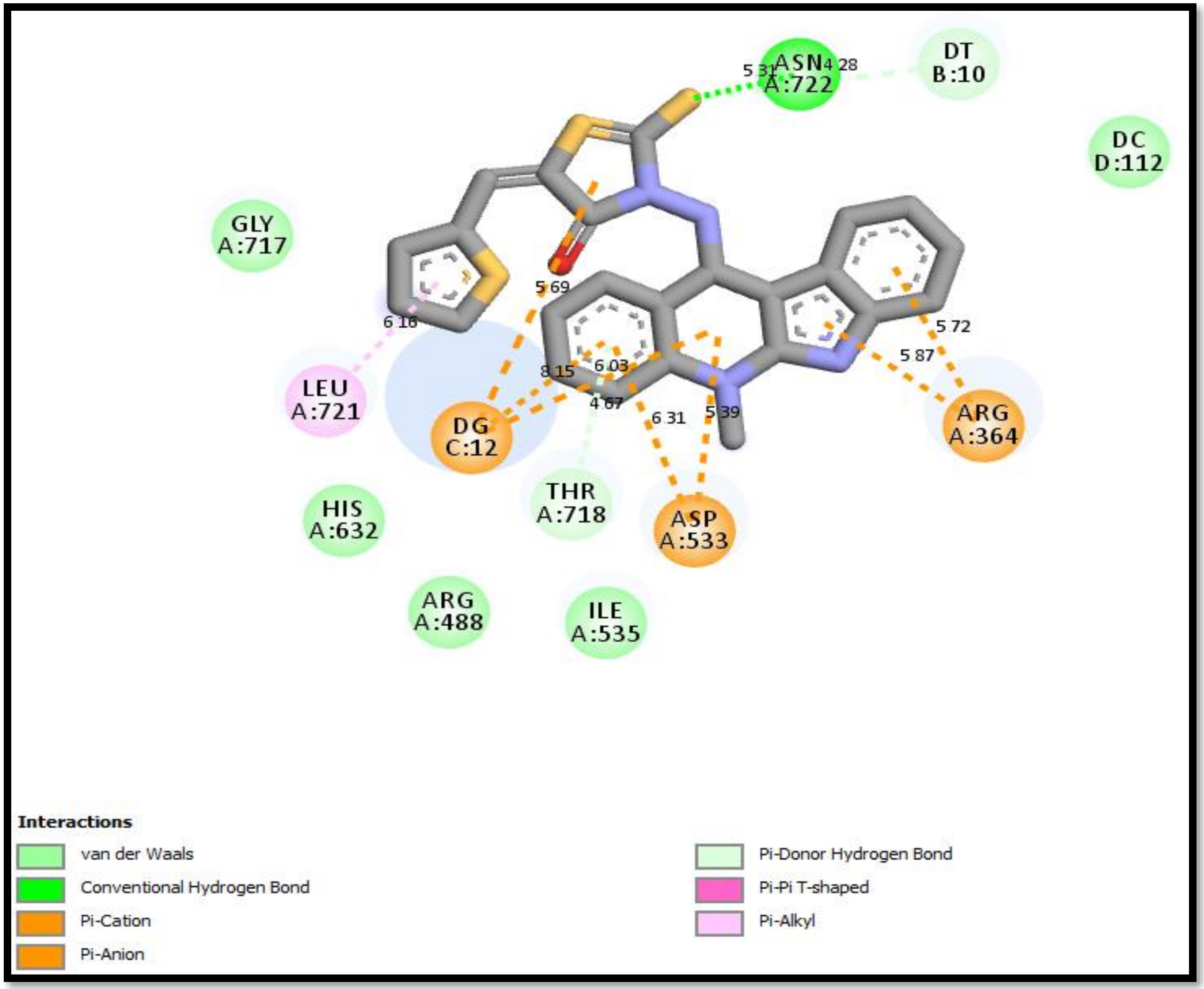

Figure S26b: docked complex of Topoisomerase I enzyme and compound **16b** interactions.

Table S7: inhibition mechanism of topoisomerase I- seven inhibitor complexes.

Compound	B. E.	Pi-cation or anion	Residues involved in hydrophobic interactions/van derWaals
**9a**	-7.51	Arg364	LYs532,Ile535,Asn631,Gln633,Asn722
**9b**	-6.5	Arg364,Asp533	Arg488,Lys532,Gly717,Thr718,Leu721,Asn722,
**11c**	-6.58	-	Asn352,Pro431,Gly717,Thr718
**11d**	-5.9	Asp533	Arg364,Arg488,Ile535,Gly717,Thr718, Lys751
**14**	-6.86	-	Arg364,Pro431,Gly717,Thr718,Leu721,Lys751
**16a**	-7.34	Asp533	Ile535,Gly717,Thr718,Leu721,Asn722
**16b**	-7.77	Arg364, Asp533	Ile535,Gly717,Thr718,Leu721,Asn722

B.E. equal to Binding Energy (Kcal/mol)

Table S7: continued

Compound	pi-sigma	H-bond	Pi-alkyl, Alkyl	Pi-sulfer	Amide-pistacked
**9a**	-	Arg488,Asp533, Thr718	-	His632	-
**9b**	-	-	His632	-	-
**11c**	Leu721	Asn722	-	-	-
**11d**	-	-	Leu721	-	-
**14**	-	Asn722	-	-	-
**16a**	-	Arg364	Leu721	-	-
**16b**	-	-	Leu721	-	-

H-bond refers to hydrogen bond.

Table S8: Calculations of physical properties for the seven compounds.

Ligand	Log P	TPSA	N.atoms	MW	nON	nOHNH	nv	Nr	volume
**9a**	-4.93	58.65	28	407.54	5	2	0	4	346.78
**9b**	-4.73	65.12	38	547.77	7	2	1	9	495.92
**11c**	-2.88	65.12	45	637.9	7	2	1	11	584.13
**11d**	-3.36	85.35	46	653.9	8	3	1	11	592.15
**14**	-4.94	58.65	26	379.49	5	2	0	2	313.18
**16a**	-3.2	58.65	34	487.61	5	2	0	4	406.32
**16b**	-3.47	58.65	32	475.64	5	2	0	4	392.1

TPSA: total molecular polar surface area. nON: Hydrogen bond acceptor; nOHNH: hydrogen bond donor nv: number of violations, Nr: number of rotatable bonds.

Table S9: Hydrophobic interactions of the seven compounds.

Ligand	H-bonds	S(L/L)	S(H/H)	Sburied	Stotal	Match1	Match[2]	Stack.	Stack. Gua-Pi
**9a**	0.03	176.90	0.00	227.59	308.03	0.5743	0.7296	1.17	1.44
**9b**	0.55	242.02	2.91	290.94	405.94	0.6034	0.7888	1.02	1
**11c**	2.53	271.51	3.10	252.34	425.36	0.6456	0.8041	0.84	0
**11d**	0.91	330.12	6.52	323.41	512.01	0.6575	0.8127	1.09	0.9
**14**	0.20	167.34	0.00	183.60	271.83	0.6156	0.7621	1.54	0
**16a**	0.01	214.81	0.00	229.17	347.40	0.6183	0.7642	2.06	0.37
**16b**	0.00	181.26	0.00	249.74	341.06	0.5315	0.6941	1.2	1.28

Table S10: Calculation of bioactivity scores for compounds 9a-16b.

No.	GPCRL	ICM	KI	NRL	PI	EI
**9a**	-0.01	0.35	0.05	-0.97	-0.28	0.27
**9b**	0.09	0.23	0.03	-0.82	-0.17	0.25
**11c**	-0.11	-0.47	-0.34	-1.08	-0.2	-0.13
**11d**	-0.16	-0.58	-0.43	-1.1	-0.23	-0.2
**14**	-0.07	0.23	0.06	-0.92	-0.26	0.22
**16a**	0.01	0.12	0.11	-0.63	-0.18	0.22
**16b**	-0.09	0.07	0.03	-0.84	-0.25	0.17

GPCRL: GPCR ligand; ICM: ion channel modulator; KI: kinase inhibitor; NRL: nuclear receptor ligand, PI: protease inhibitors; EI: enzyme inhibitor.
